# Substrate recognition by a peptide-aminoacyl-tRNA ligase

**DOI:** 10.1073/pnas.2423858122

**Published:** 2025-03-19

**Authors:** Josseline Ramos-Figueroa, Haoqian Liang, Wilfred A. van der Donk

**Affiliations:** ^a^Department of Chemistry, University of Illinois at Urbana-Champaign, Urbana, IL 61801; ^b^HHMI, University of Illinois at Urbana-Champaign, Urbana, IL 61801; ^c^Department of Biochemistry, University of Illinois at Urbana-Champaign, Urbana, IL 61801

**Keywords:** peptide bond, amino acyl-tRNA, conjugation

## Abstract

The continuing discovery of new peptide-aminoacyl-tRNA ligases (PEARLs) has unveiled a diverse array of enzymes with the unique potential to append amino acids to the C terminus of substrate peptides in an aminoacyl-tRNA-dependent manner. To date, PEARLs have been reported that can conjugate Cys, Ala, Trp, Gly, Leu, Asn, and Thr residues, but the basis of peptide substrate and aminoacyl-tRNA recognition is not known. Cell-free expression (CFE) has emerged as a powerful tool to rapidly assay activity of substrate variants, and we used the technique in this study to investigate the peptide substrate specificity of the PEARL ${BhaB}_{C}^{Trp}$. This enzyme that adds Trp was discovered previously during genome mining for ribosomally synthesized and posttranslational modified peptides (RiPPs). The enzyme is remarkably tolerant of changes to the C-terminal amino acid of the peptide substrate, and truncation and replacement experiments suggest a minimal sequence requirement. An AlphaFold3 model provided insights into binding interactions of the substrate peptide BhaA-Ala to ${BhaB}_{C}^{Trp}$ and also generated predictions for tRNA, ATP, and Mg^2+^ binding modes that were tested by site-directed mutagenesis. The data suggest that several highly conserved residues in PEARLs recognize the 3′-CCA sequence present in all tRNAs. The minimal sequence required for Trp incorporation by ${BhaB}_{C}^{Trp}$ was employed as a protein tag for C-terminal labeling of eGFP, lysozyme, and MBP with Trp and 5-Br-Trp.

## Significance

Peptide synthesis in nature is mostly accomplished by the ribosome or nonribosomal synthetases. Recently, a class of enzymes was discovered termed peptide aminoacyl-tRNA ligases (PEARLs) that use aminoacyl-tRNA to attach the amino acid to the C terminus of peptides. Given the importance of amide bond formation for the preparation of natural and non-natural peptides, this study investigated the substrate specificity of a representative PEARL. The enzyme is tolerant of a wide range of amino acids at the C terminus of the peptide substrate, providing avenues for engineering the enzyme to prepare designer molecules. Identification of a minimal substrate sequence and appendage of this sequence to various proteins allowed the conjugation of tryptophan and 5-bromo-tryptophan to their C termini.

**Fig. 1.**
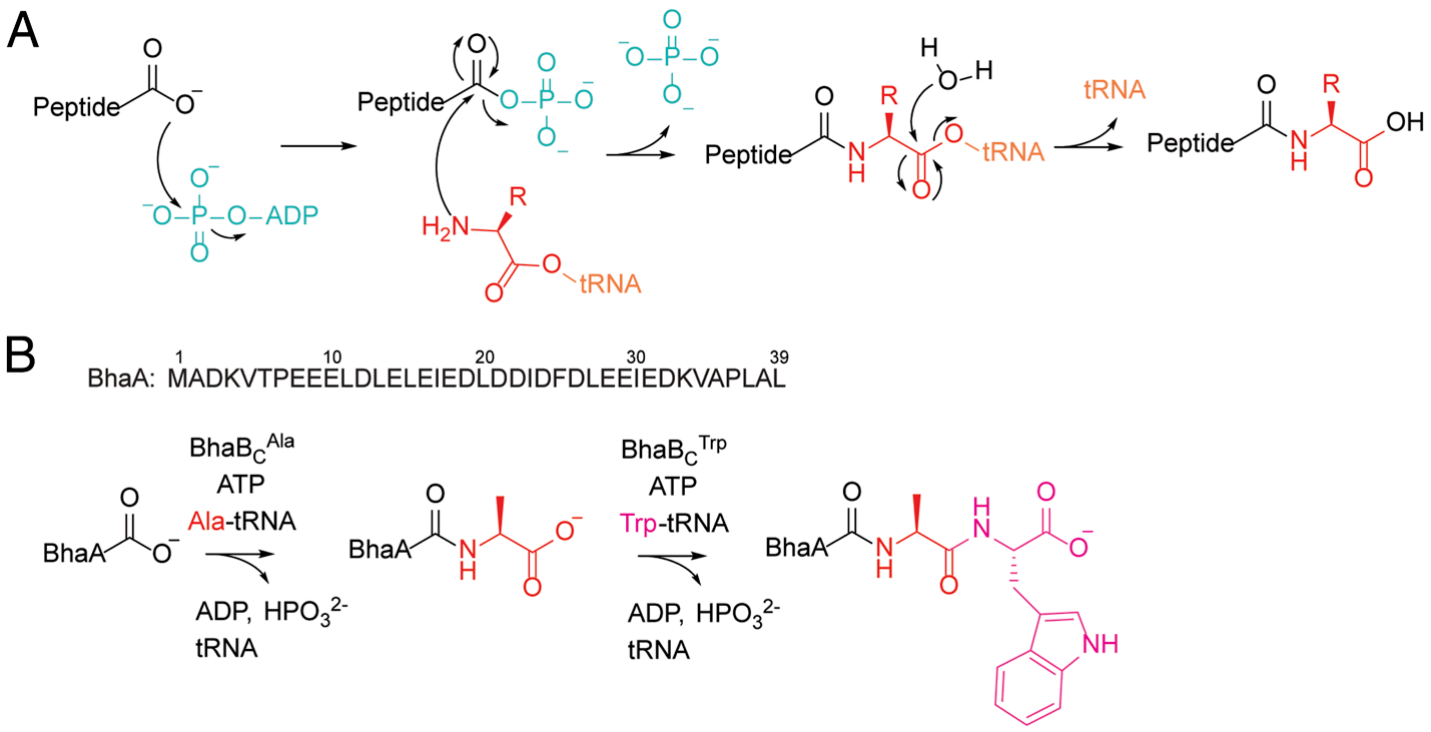
(*A*) Mechanism of nonribosomal peptide extension by PEARLs. (*B*) BhaA sequence and the cascade reactions catalyzed by ${BhaB}_{C}^{Ala}$ and ${BhaB}_{C}^{Trp}$ on BhaA.

Techniques that enable the labeling of proteins have extensive applications in biomolecular research (1–7). The integration of noncanonical amino acids (ncAAs) provides proteins with distinctive bioorthogonal handles for conjugation with exogenous molecules, facilitating investigation of biological processes in vitro and in living organisms (8–10). To date, the introduction of ncAAs into protein is mostly achieved through orthogonal translation systems, in which orthogonal tRNA and aminoacyl-tRNA synthetase pairs are engineered or recoded to incorporate ncAAs at specific codons in the mRNA by the ribosome (11–17). Despite the power of this method, incompatibility of orthogonal components with the ribosome can lead to miscoding and decrease in incorporation efficiency (18). Nature offers potential alternative strategies using the diverse array of enzymes discovered in the biosynthetic pathways of peptide-derived natural products. For instance, a recently discovered class of enzymes enables amino acid appendage to peptides independent of the ribosome, making them promising candidates for ncAA incorporation through further bioengineering. These enzymes, termed peptide aminoacyl tRNA ligases (PEARLs), use amino acyl-tRNA as amino acid donor and catalyze the conjugation of the charged amino acid to the C terminus of a substrate precursor peptide in an ATP-dependent and template-independent manner (19–22). During the reaction, PEARLs utilize ATP to first phosphorylate the C-terminal carboxylate of their peptide substrates. Next, the activated carboxyl group is condensed with the amino group of aminoacyl-tRNA, forming a new amide bond (Fig. 1*A*) (19, 20). Hydrolysis of the tRNA then provides a peptide that has been extended by one amino acid. A collection of different PEARLs have been demonstrated to incorporate a wide range of amino acids into their substrates (19–21, 23, 24).

PEARLs are evolutionarily related to class I lanthipeptide dehydratases (LanBs) (25), enzymes that glutamylate the side chains of Ser and Thr residues in their substrate peptides in a glutamyl-tRNA-dependent mechanism. The glutamate is then eliminated by the LanB C-terminal elimination domain, resulting in the formation of dehydroamino acids (26). In some cases, including enzymes involved in the biosynthesis of thiopeptides and pyritides (27–29), the glutamylation and elimination domains are two separate proteins, resulting in the distinction between LanB_A_ proteins (single enzymes) and LanB_B_ proteins (separate glutamylation and elimination proteins) (21). PEARLs typically only contain the domain that resembles the LanB glutamylation domain (20, 26) and lack the elimination domain; these enzymes have been designated ${LanB}_{C}^{Xxx}$, with Xxx indicating which amino acid is appended to the substrate peptide from amino acyl-tRNA. A major difference between PEARLs and LanB_A/B_ enzymes is that the former require ATP whereas the latter do not. A recent bioinformatic analysis (23) provided support for a model in which PEARLs evolved from ATP-GRASP proteins and that LanB_A/B_ proteins further evolved and lost the ATP requirement. At present, no information is available for PEARLs or LanBs regarding the binding site of the aminoacyl-tRNA nor is it known where the active site for amide bond formation is located.

Despite the diverse bacterial origins of PEARL-encoding biosynthetic gene clusters (BGCs), thus far, all identified PEARLs from different phyla have exhibited activity with aminoacylated tRNA substrates in *Escherichia coli*, suggesting a relaxed specificity regarding the tRNA sequence. This flexibility also illustrates their potential compatibility with orthogonal translation components and positions them as promising candidates for introducing ncAAs into target peptides or proteins. Furthermore, the process of incorporating an amino acid at the end of a growing peptide and then exposing a new terminus for subsequent coupling to the next amino acid (Fig. 1*B*) has conceptual similarities to solid phase peptide synthesis (SPPS). Given the recent rise of peptides as a drug modality and the high chemical waste associated with SPPS (30), enzymatic means of synthesizing peptides in a nontemplated manner hold significant potential. Effective application of PEARLs for amino acid appendage to non-native peptides or proteins requires a comprehensive understanding of substrate recognition. Thus, in this study, we set out to investigate the mechanism of substrate recognition by a representative PEARL enzyme through site-directed mutagenesis of the substrate peptide.

Cell-free expression (CFE) is emerging as a powerful technique to rapidly probe the importance of specific residues in substrate recognition (31). In CFE, proteins are synthesized from input DNA templates using cell lysates or purified components that contain the necessary cellular machinery for transcription and translation (*SI Appendix,* Fig. S1) (31–33). This approach offers several advantages, including speed, absence of nucleases and proteases that potentially lead to low expression yield, and the ability to synthesize proteins (or RiPP products) that may be toxic or difficult to express in living cells (34–36). Indeed, CFE has recently been applied in several RiPP biosynthetic investigations (29, 37–41). For instance, CFE-based activity assessment of the enzymes involved in the biosynthesis of thiopeptides, pyritides, and lasso peptides comprehensively investigated their substrate tolerance within a relatively short timeframe (29, 38, 39, 42–45), and discovery of novel lantibiotics was achieved through CFE-assisted development of libraries of nisin and lacticin 481 analogues (37, 41).

In this study, we utilized CFE methodology to investigate the substrate recognition mechanism of ${BhaB}_{C}^{Trp}$, a PEARL that adds Trp from tryptophanyl-tRNA to the C terminus of its substrate peptide (Fig. 1*B*) (21). Trp is the most rare amino acid in proteins and can function as a handle for site-selective modification (46, 47). ${BhaB}_{C}^{Trp}$ is encoded in a BGC from *Bacillus halodurans* C-125, *bha*, believed to produce a pyrroloquinoline-like natural product given its similarity to the ammosamide BGC (21). In the first biosynthetic step, the PEARL ${BhaB}_{C}^{Ala}$ adds Ala to the C terminus of the precursor peptide BhaA forming BhaA-Ala, then ${BhaB}_{C}^{Trp}$ installs a Trp to the C terminus of BhaA-Ala producing BhaA-Ala-Trp (Fig. 1*B*). The CFE experiments in this study demonstrate that ${BhaB}_{C}^{Trp}$ displays considerable tolerance toward variations in the BhaA-Ala sequence including substitution of the C-terminal residue, Ala40 (Fig. 1*B*), with polar, nonpolar, bulky, and small amino acids. Using AlphaFold3 (48), we generated a model of the quaternary complex of ${BhaB}_{C}^{Trp}$, tRNA^Trp^, BhaA-Ala, and ATP providing a three-dimensional perspective of peptide substrate and tRNA engagement by the enzyme. We identified residues in ${BhaB}_{C}^{Trp}$ that were predicted to interact with the 3′ CCA sequence and the acceptor stem of tRNA and provide support for the model through mutagenesis studies. Furthermore, by evaluating the activity of ${BhaB}_{C}^{Trp}$ toward truncated BhaA substrates and the AlphaFold3 model, a minimal sequence was obtained for Trp incorporation that was used as a tag at the C termini of eGFP, lysozyme, and MBP and that also allowed incorporation of 5-Br-Trp at the C terminus of these proteins.

## Results and Discussion

### ${\text{BhaB}}_{C}^{\text{Trp}}$ Fully Modifies BhaA-Ala Generated by CFE.

**Fig. 2.**
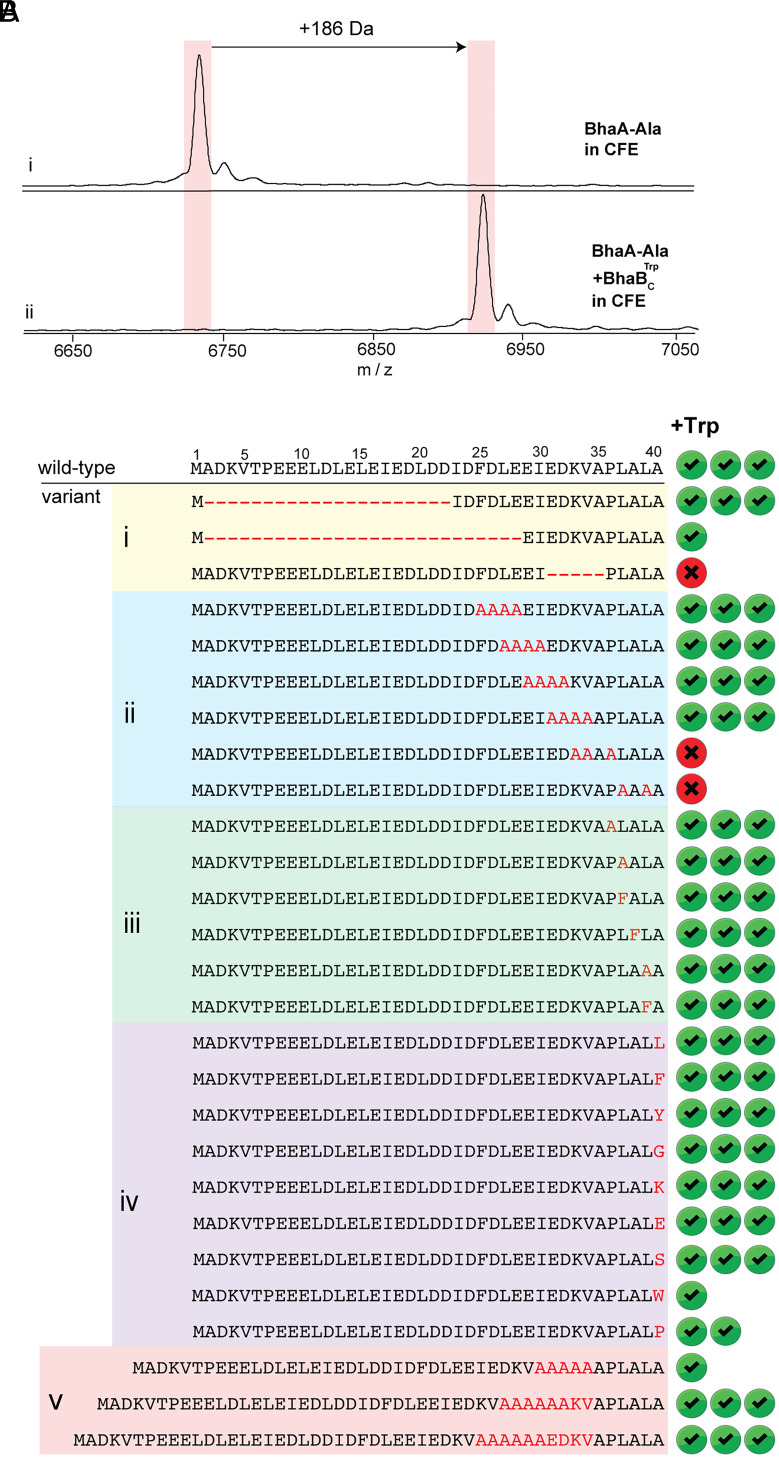
Activity and substrate specificity assessment of ${BhaB}_{C}^{Trp}$ made by CFE. MALDI-TOF MS analysis of (*A*) CFE of (*i*) His_6_–BhaA-Ala. [fMet + M + H]^+^ calcd. *m/z* = 6733.2, obsd. *m/z* = 6734.7. (*ii*) CFE of substrate along with ${BhaB}_{C}^{Trp}$. (fMet + M + H + Trp – H_2_O)^+^ calcd. *m/z* = 6919.3, obsd. *m/z* = 6921.5. (*B*) Summary of ${BhaB}_{C}^{Trp}$ activity toward Bha-Ala variants including (*i*) truncated substrates, (*ii*) block-wise Ala replacements, (*iii* and *iv*) single-site variants, and (v) sequence extensions to evaluate changes in register of the C-terminal PLALA sequence. Three check marks indicate >90% conversion, two check marks ~50% conversion, and one check mark indicates product is formed but more than 50% of the starting peptide remained in at least two independent duplicate experiments under identical conditions (*SI Appendix,* Figs. S3–S8). A cross indicates no conversion.

In most previous studies that used CFE to investigate the substrate specificity of RiPP biosynthetic enzymes, variants of the substrate peptide were produced by CFE, and then, purified enzyme was added. In only a few cases, the enzymes themselves were also produced by CFE (37, 39, 41). To establish proof-of-concept for the feasibility of using CFE to probe the substrate requirement of ${BhaB}_{C}^{Trp}$, expression of both BhaA-Ala and ${BhaB}_{C}^{Trp}$ was evaluated using the PURExpress**^®^** in vitro protein synthesis system (34). Pairwise CFE reactions were set up containing plasmid encoding hexahistidine (His_6_) tagged BhaA-Ala or plasmids encoding both the peptide substrate and ${BhaB}_{C}^{Trp}$. The reactions were monitored by matrix-assisted laser desorption/ionization time-of-flight (MALDI-TOF) mass spectrometry (MS). Additionally, both reaction mixtures were analyzed by SDS-PAGE revealing a unique band corresponding to ${BhaB}_{C}^{Trp}$ (*SI Appendix,* Fig. S2). A peak corresponding to the mass of His_6_-tagged BhaA-Ala was detected in the MALDI-TOF mass spectrum when substrate was expressed alone, and a shift in mass consistent with the condensation of a Trp was observed when enzyme and substrate were coexpressed, indicating ${BhaB}_{C}^{Trp}$ produced by CFE transformed the substrate BhaA-Ala into a product bearing a Trp appendage (Fig. 2*A*).

### A C-Terminal 12-mer of BhaA-Ala Is Sufficient for Modification by ${\text{BhaB}}_{C}^{\text{Trp}}$.

The activity of ${BhaB}_{C}^{Trp}$ in the CFE experiments prompted a detailed study into the features in the substrate peptide that are important for processing by the enzyme. To investigate the minimal requirement of the substrate length for ${BhaB}_{C}^{Trp}$, two shortened variants of BhaA-Ala were designed (Fig. 2 *B*, panel *i*). DNA encoding these peptide variants were acquired as double-stranded DNA fragments containing a T7 promoter, ribosome binding site (RBS), and each corresponding open reading frame (ORF) linked by spacers (*SI Appendix,* Fig. S3) (29). These DNA fragments were then combined with the plasmid encoding ${BhaB}_{C}^{Trp}$ for expression using PURExpress®. The assay mixtures were desalted and analyzed by MALDI-TOF MS. ${BhaB}_{C}^{Trp}$ catalyzed the extension of an 18-mer peptide truncant spanning Ile23 to Ala40. Conjugation of Trp to the C terminus of a C-terminal 12-mer peptide (from Glu29 to Ala40) was also observed albeit to a much lesser extent as judged by the low intensity signal of the +186 Da peak with respect to the mutant substrate peak (*SI Appendix,* Fig. S4). These results indicated that the residues from the N-terminus to Glu28 are not critical for the enzyme–substrate interaction, but that residues in the region from Ile23 to Glu28 are important for optimal processing by ${BhaB}_{C}^{Trp}$.

### Ala Scanning to Investigate Substrate Specificity.

To identify essential residues in BhaA-Ala for recognition by ${BhaB}_{C}^{Trp}$, blockwise Ala scanning of the C-terminal region of the peptide was performed (panel *ii*, Fig. 2*B*). Consistent with the previous sequence deletion data where residues Ile23 to Glu28 were not critical for activity, ${BhaB}_{C}^{Trp}$ added Trp to a variant where Phe25 to Glu28 (sequence FDLE) were replaced by Ala residues (Fig. 2*B* and *SI Appendix,* Fig. S5 and Table S2). ${BhaB}_{C}^{Trp}$ fully converted this mutant to the corresponding product, whereas the deletion mutant where these residues were completely removed was only partially processed. This result suggests that ${BhaB}_{C}^{Trp}$ is sensitive to the length of the peptide substrate, possibly for the peptide to attain secondary structure. In a similar manner, ${BhaB}_{C}^{Trp}$ also added Trp to variants in which a subset of four sequential amino acids from Leu27 to Val34 were substituted with Ala residues (Fig. 2, panel *ii*, and *SI Appendix,* Fig. S5). However, when the C-terminal PLALA sequence was changed to PAAAA, ${BhaB}_{C}^{Trp}$ was not able to process the peptide variant. Previous bioinformatic analysis showed that the C-terminal PLALA sequence of BhaA-Ala is highly conserved among several PEARL substrate peptides from different phyla (19, 21). ${BhaB}_{C}^{Trp}$ was also unable to conjugate Trp to the end of a peptide in which the sequence preceding the LALA motif (Lys33 to Pro36) was substituted by Ala residues (Fig. 2*B* and *SI Appendix*, Fig. S5 and Table S2). Similarly, deletion of the EDKVA sequence abolished activity (Fig. 2*B*). Collectively, the deletion and Ala-block scanning experiments indicated that ${BhaB}_{C}^{Trp}$ is tolerant of changes up to roughly Pro36, but that substitutions beyond that point appear to disrupt activity.

We next attempted to determine whether specific residues in the C-terminal five residues are critical for activity. We first focused on the two Leu residues (Leu37 and Leu39, panel *iii*, Fig. 2*B*). Starting from the inactive PAAAA variant, reintroduction of either Leu37 or Leu 39 was sufficient to restore Trp conjugation activity (*SI Appendix,* Fig. S6). Activity was not specific to Leu residues as ${BhaB}_{C}^{Trp}$ was also active when Phe substitutions were introduced at positions 37 and 39 (Fig. 2*B*). Furthermore, Ala38 was not a critical residue as substitution with Phe still resulted in a substrate that was processed by ${BhaB}_{C}^{Trp}$. We also explored the importance of Pro36 for activity and found that replacement with Ala resulted in a substrate for ${BhaB}_{C}^{Trp}$. These findings show that the enzyme is quite forgiving in terms of individual substitutions and suggest that it may be a secondary structural feature that is recognized. A discussion based on AlphaFold3 models is provided in a later section of this study.

### ${\text{BhaB}}_{C}^{\text{Trp}}$ Is Tolerant of Changes in the C-Terminal Residue of BhaA-Ala.

The C-terminal Ala of the LALA motif was investigated next with a series of single-site variants (panel *iv*, Fig. 2*B*). ${BhaB}_{C}^{Trp}$ conjugated Trp to all investigated variants in which Ala40 was substituted with a wide array of amino acids, including polar or charged residues (Ser, Lys, and Glu) and hydrophobic and aromatic residues (Leu, Phe, Trp, and Tyr) (Fig. 2*B* and *SI Appendix,* Fig. S7 and Table S2). ${BhaB}_{C}^{Trp}$ also added Trp to the carboxyl group of Pro40 and Gly40, amino acids with distinct conformational preferences. Of these variants, A40P and A40W were only partially processed by the enzyme. The relatively low specificity for the C-terminal residue suggests that the size, polarity, or charge of the side chain of the C-terminal residue is not critical for recognition and again suggests that secondary structure and/or substrate length may be important.

Last, we made extended substrates in which Ala blocks were inserted before Ala35 (BhaA-Ala extend-1), Lys33 (BhaA-Ala extend-2), and Glu31 (BhaA-Ala extend-3, panel *v*, Fig. 2*B*). When these variants were used in the CFE assays, ${BhaB}_{C}^{Trp}$ added Trp to all peptides as shown by MALDI-TOF MS analysis (Fig. 2*B* and *SI Appendix,* Fig. S8 and Table S2).

### AlphaFold3 Predictions of the BhaA-Ala:${\text{BhaB}}_{C}^{\text{Trp}}$:tRNA^Trp^:ATP Quaternary Complex.

To rationalize the results of the CFE studies and obtain insights into the potential mechanism of tRNA recognition, we employed the recently launched AlphaFold3 program with its added feature of ligand modeling including RNA (48, 49). In addition to BhaA-Ala, we included ATP, Mg^2+^ ions, and tRNA^Trp^ from *E. coli,* but without posttranscriptional modifications since these are not currently supported. AlphaFold-based algorithms predict the structure of wild-type (WT) proteins based on covariance of amino acid sequences. Assessing the confidence in AlphaFold3-based predicted complexes involves analysis of two parameters, the predicted local distance difference test (pLDDT) and the predicted aligned error (PAE). Compared to earlier versions that determined a pLDDT value as a per-residue measure of local confidence, AlphaFold3 predicts these values for each atom in the predicted structure. Regions with pLDDT values larger than 90 are regarded as high-confidence predictions that can be used for binding analysis including the side chains of residues. pLDDT values higher than 70 but lower than 90 represent good confidence suggesting correct backbone prediction. pLDDT values lower than 70 are not reliable (50). In addition, PAE values represent the level of confidence on the relative position of two amino acid residues within the predicted structure. When these residues are present in two different domains or objects of a complex, high PAE values indicate that the relative position between these two amino acids cannot be predicted confidently. In our evaluation of the predicted quaternary structure, we used both pLDDT and PAE values. We will show only the top model in the discussion below, but the top five structures were highly similar (*SI Appendix,* Fig. S9).

**Fig. 3.**
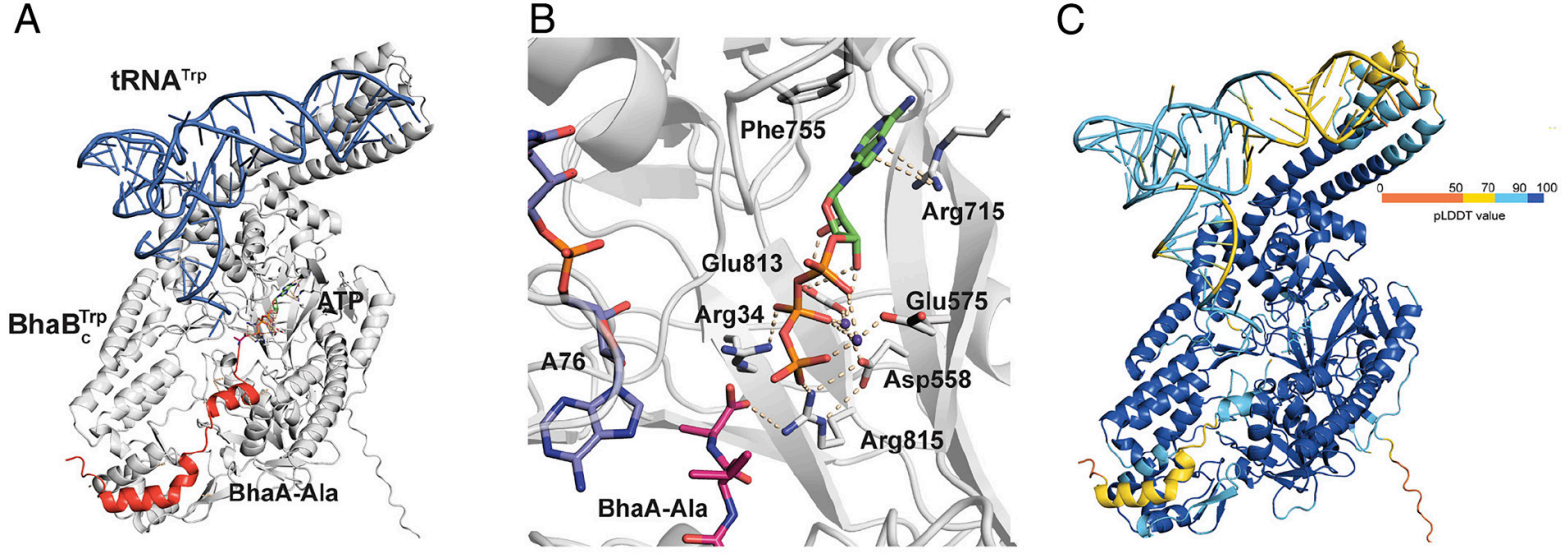
(*A*) General view of the quaternary complex comprising ${BhaB}_{C}^{Trp}$:tRNA^Trp^:BhaA-Ala:ATP predicted by AlphaFold3. The enzyme is in gray, tRNA in blue, and substrate peptide in red. (*B*) Predicted ATP binding to the postulated ATP-GRASP domain in ${BhaB}_{C}^{Trp}$. Key interactions are shown with wheat-colored dashes including predicted polar interactions between the BhaA-Ala C-terminal carboxylate and Arg815 and Arg34 of ${BhaB}_{C}^{Trp}$. BhaA-Ala residues are shown as pink sticks and the carbon atoms of the 3′ adenosine (A76) are shown in marine blue sticks. (*C*) Quaternary complex shown in a pLDDT-based coloring scheme. Dark blue corresponds to pLDDT values higher than 90, light blue to pLDDT scores higher than 70 but less than 90, yellow to pLDDT values less than 70 but higher than 50, and low pLDDT scores (<50) are shown in orange.

The binding of tRNA to LanB and PEARL enzymes has remained elusive as no structures have been reported. It was therefore striking that AlphaFold3 places the 3′-end of the tRNA^Trp^ molecule right into the putative active site of ${BhaB}_{C}^{Trp}$ based on previous mutagenesis studies on the Cys-adding PEARL TglB (Fig. 3*A*) (20). A recent bioinformatic analysis of PEARLs concluded that they contain an ATP-GRASP-like ATP binding site that is involved in activation of the C-terminal carboxylate group of its peptide substrate by phosphorylation (23). In line with this prediction, AlphaFold3 positioned the ATP together with two Mg^2+^ ions in the postulated ATP-GRASP binding site making several interactions with ${BhaB}_{C}^{Trp}$ residues (Glu813, Arg815, and Asp558) that have already been shown experimentally to be important for the phosphorylation step catalyzed by TglB (Glu801, Arg803, and Glu542) (20, 23). Finally, the model also placed the C-terminal carboxylate of the peptide substrate right next to the γ-phosphate of ATP (Fig. 3*B*). Hence, in terms of the chemistry that ${BhaB}_{C}^{Trp}$ catalyzes, the predicted quaternary complex is primed for catalytic activity.

The confidence in the predicted complex structure was assessed using the pLDDT (*SI Appendix,* Fig. S10) and PAE values (*SI Appendix,* Fig. S11). The quaternary complex is shown in Fig. 3*C* colored by pLDDT, illustrating good to high confidence in the model in key areas of interaction of the enzyme with both the peptide substrate and certain parts of the tRNA substrate. Individual pLDDT plots for enzyme, tRNA, and peptide substrate in the top five models are shown in *SI Appendix,* Fig. S10. For all five models, most of the tertiary structure of ${BhaB}_{C}^{Trp}$ is predicted with good to high confidence (pLDDT > 80), with low confidence regions (50 < pLDDT < 70) for the first N-terminal 23 amino acids, and for residues at positions 78 to 87 and 105 to 116. PAE plots are shown in *SI Appendix,* Fig. S11; as mentioned, the PAE values measure the relative confidence between any two residues at any part of the complex. ChimeraX (51) was used to visually evaluate the confidence of interactions at the interface between the individual components of the quaternary complex involving ${BhaB}_{C}^{Trp}$, BhaA-Ala, ATP, and tRNA^Trp^ (*SI Appendix,* Fig. S11*C*). Low PAE values (<5 Å) were observed for interactions of the enzyme with the C-terminal helix of BhaA-Ala and with ATP, with slightly larger PAE values for the interactions between ${BhaB}_{C}^{Trp}$ and the 3′ CCA sequence of tRNA^Trp^. Significantly larger PAE values were observed for putative interactions with the anticodon region. Below we will discuss in turn the predicted interactions with ATP, tRNA, and the peptide substrate.

### AlphaFold3 Predictions of the Interaction of ${\text{BhaB}}_{C}^{\text{Trp}}$ with ATP.

All enzyme residues embedded in the ATP-GRASP-like fold were predicted with high confidence (pLDDT values > 90), and as such, the side chain residue interactions with ATP within this fold are further discussed. In the model, Glu813 interacts through hydrogen bonds with the 2′ and 3′ hydroxyl groups of the ribose moiety, and with one of the two Mg^2+^ ions (Fig. 3*B*) that coordinates to the β-phosphate oxygens. In addition, Asp558 coordinates to the same Mg^2+^ ion and interacts with Arg815, which in turn engages the γ phosphate oxygens of ATP and the C-terminal carboxylate of BhaA-Ala. A second Mg^2+^ ion is predicted to be coordinated by Glu575 and possibly the α, β, and γ phosphates of ATP. As noted above, several of the corresponding residues of the PEARL TglB were previously shown to be important for phosphorylation of its peptide substrate (20), providing further support for the predicted binding model.

The side chains of Arg715 and Phe755 are in close proximity to the adenine group of ATP with Arg715 likely interacting with the adenine moiety through a cation–pi interaction. This interaction is reminiscent of a previously observed interaction in the cocrystal structure of TbtB, a glutamyl-tRNA-dependent LanB_B_ enzyme, coordinated to an AMP analog that carried a glutamate on the 2′-amino group (52). TbtB catalyzes a different reaction from that catalyzed by PEARLs and does not utilize ATP as substrate. However, the X-ray structure displays a very similar interaction of the adenine moiety of the glutamyl-tRNA analog with an Arg and Phe as the interaction observed in the AlphaFold3 model of ${BhaB}_{C}^{Trp}$ involving Arg715 and Phe755 and the adenine of ATP.

### AlphaFold3 Predictions of the Interaction of ${\text{BhaB}}_{C}^{\text{Trp}}$ with tRNA^Trp^.

**Fig. 4.**
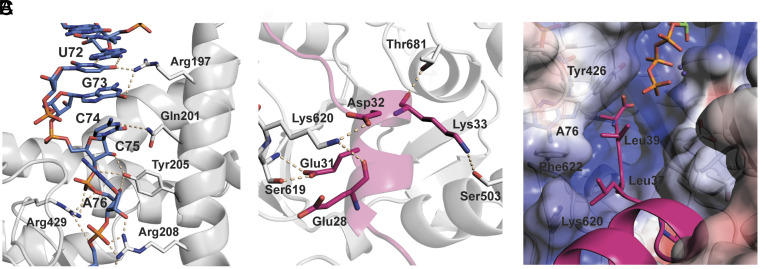
Predicted interactions between ${BhaB}_{C}^{Trp}$ and its peptide and tRNA substrates. (*A*) Predicted binding interactions of the 3′ CCA end of the tRNA^Trp^ with residues in ${BhaB}_{C}^{Trp}$. tRNA bases are shown in blue sticks, and enzyme residues with white gray sticks. Polar interactions are indicated with wheat-colored dashes. (*B*) Predicted binding mode of BhaA-Ala (pink) and interactions with ${BhaB}_{C}^{Trp}$ residues (white gray). (*C*) Predicted binding mode of the C-terminal PLALA motif of BhaA-Ala (in pink) bound to a groove in ${BhaB}_{C}^{Trp}$ that leads to the ATP binding site. Electrostatic potential surface of the enzyme is shown with white patches indicating hydrophobic regions, blue patches positively charged regions, and red patches negatively charged zones.

The tRNA^Trp^ molecule in all models is overall of lower confidence but still mostly with pLDDT values >60, with higher pLDDT scores (80 to 90) for the T- and D-arm regions (base positions 43 to 65) and the 3′-CCA end (base positions 73 to 76) (Fig. 3*C* and *SI Appendix*, Fig. S10*C*). Lower pLDDT values were consistently observed for bases in the anticodon arm and loop (bases 25 to 42). The predicted structure revealed potential interactions between ${BhaB}_{C}^{Trp}$ and the tRNA^Trp^ structure, with these interactions localized at positively charged patches of the enzyme including the highly conserved Arg208, Tyr205, Gln201, and Arg197 residues (pLDDT > 90) residing in a helix comprising residues 193–211, as well as Arg429 in a loop made up of residues 415–432 (70 < pLDDT < 90). Arg208 is predicted to interact with the phosphate ester bond between nucleotides C75 and A76 of the terminal 3′-CCA sequence (70 < pLDDT < 90) in addition to the 2′ hydroxyl groups of both C75 and A76 (Fig. 4*A*). In support of an important role of Arg208 for tRNA binding, mutation of the corresponding Arg residue in a previous study of TglB resulted in complete abolishment of PEARL activity without impairing ATP-dependent activation of the C-terminal carboxylate of its substrate TglA (20). Since all tRNAs end in CCA at the 3′ end, the model explains the complete conservation of Arg208 in all experimentally characterized PEARLs even though they add a wide variety of amino acids. In the model, Tyr205 interacts with the pyranose oxygen of the ribose ring of C75, as well as a phosphate ester oxygen and the 2′ hydroxyl of C74. Gln201 is predicted to hydrogen bond with N3 of C74, and Arg197 to interact with N2 of the discriminator base G73 and with O4 of U72 (Fig. 4*A*). Finally, Arg429 is located in a proximal loop and potentially interacts with oxygens of two phosphate ester bonds of the 3′ CCA motif; however, because of the lower pLDDT values of this region, the side chain positioning is of lower confidence. Intriguingly, the anticodon loop of the bound tRNA^Trp^ appears to engage through polar interactions with a helix bundle of ${BhaB}_{C}^{Trp}$ that was previously noted as a unique part of LanB_B_ and PEARL enzymes (52) (*SI Appendix,* Fig. S12). This type of predicted interaction was also observed for AlphaFold3 models of ${BhaB}_{C}^{Ala}$ and TglB with their cognate tRNAs (*SI Appendix,* Fig. S12) suggesting it may be a general feature of PEARLs. However, the pLDDT and PAE values for enzyme and tRNA in this region are of low confidence (*SI Appendix,* Fig. S11*C*) and this potential interaction will require investigation using other tools such as the Flexizyme technology that can change the tRNA sequence without affecting charging with a desired amino acid (53).

We probed the predictions of the AlphaFold3 model by disruption of putative interactions between the enzyme and the 3′-CCA sequence of tRNA. We made R208A, Y205A, and R429A mutants of ${BhaB}_{C}^{Trp}$ and assessed their activities in the CFE assay. All three mutants showed severe decreases in the activity of ${BhaB}_{C}^{Trp}$ (*SI Appendix,* Fig. S13) indicating that despite less confident pLDDT values, the predicted interactions mediated by Arg208, Tyr205, and Arg429 appear important for catalysis. The model leaves unresolved how the amino acid attached to the tRNA may be recognized by the enzyme. A comparison of the AlphaFold3 models for the PEARLs TglB (adding Cys) and ${BhaB}_{C}^{Trp}$ shows that the area where the amino acid is attached is surrounded by flexible loops that are likely to engage with the amino acid (*SI Appendix,* Fig. S14).

### AlphaFold3 Predictions of the Interaction of ${\text{BhaB}}_{C}^{\text{Trp}}$ with Bha-Ala.

Finally, we discuss the binding of BhaA-Ala in the quaternary complex obtained with AlphaFold3. The BhaA-Ala secondary structure was predicted with good confidence (70 < pLDDT < 90) for the C-terminal residues from positions 27 to 39 with significantly lower scores toward the N-terminus of the peptide (Fig. 3*C* and *SI Appendix*, Fig. S10*C*). As mentioned, AlphaFold3 placed the C terminus of the BhaA-Ala peptide right into the same area as the 3′-end of the tRNA^Trp^ and the γ-phosphate of ATP (Fig. 3*B*). Although this region had lower pLDDT values for residues on ${BhaB}_{C}^{Trp}$ (<70) (*SI Appendix,* Fig. S10*B*), it contains several completely conserved amino acid residues. In the model, Arg815 of ${BhaB}_{C}^{Trp}$ makes an electrostatic interaction with the C-terminal carboxylate of BhaA-Ala to likely position it for phosphoryl transfer. In agreement with the model, the corresponding residue in TglB (Arg803) was shown to be important for the phosphorylation of its substrate peptide. Arg34 is also located in the vicinity of the C-terminal carboxylate of BhaA-Ala and the ATP β phosphate, with one of the side chain guanidinium nitrogens positioned 3.2 Å from the C-terminal carboxylate while the adjacent nitrogen is 2.9 Å from the β phosphate oxygen (Fig. 3*B*). The corresponding residue in TglB (Arg10) was previously identified as a residue important for Cys appendage to its substrate TglA, but as nonessential for the phosphorylation step (20). Hence, the residue may be more important for activation of the amino acid attached to tRNA or the hydrolysis of tRNA. AlphaFold3 currently cannot use amino acyl-tRNA and thus determination of the precise role of Arg34/Arg10 will require structural biology approaches.

In addition to the interactions in the active site, several other interactions are predicted between BhaA-Ala and ${BhaB}_{C}^{Trp}$ (Fig. 4*B*). The predicted conformation of BhaA-Ala features two helical segments, from Glu8 to Asp21 and from Leu27 to Val34. The pLDDT values show a much higher confidence level for the C-terminal helix than for the N-terminal helix (pLDDT < 70, Fig. 3*C* and *SI Appendix*, Fig. S10*B*). Furthermore, the deletion mutants in the CFE studies showed that the segment of Bha-Ala that contains the N-terminal helix is dispensable for activity. Therefore, we focus here solely on the predicted interactions of ${BhaB}_{C}^{Trp}$ with the C terminus of BhaA-Ala, with a discussion of its N-terminus in the *SI Appendix*. We do note that the predicted conformation of BhaA-Ala does not result in any interactions with the RiPP recognition element (RRE) (54) that is present in ${BhaB}_{C}^{Trp}$ (see discussion in *SI Appendix*). Consistent with the possibility that the RRE may not be important for substrate engagement, the segment of the substrate peptide that would have been expected to bind to the RRE (residues 25–28) could be deleted or replaced by four Ala residues without loss of activity, an observation that was also made for another PEARL (TglB) (20).

Several interactions are predicted between ${BhaB}_{C}^{Trp}$ and the C-terminal helical segment of BhaA-Ala (Leu27 to Val34), with the Glu31 side chain on BhaA-Ala interacting through a hydrogen bond with Ser619 of the enzyme, the Asp32 side chain and Glu28 main chain carbonyl of the peptide interacting with Lys620 on the enzyme, and the Lys33 side chain of the substrate forming a hydrogen bond with Ser503 of the protein and its main chain carbonyl interacting with the nearby Thr681 side chain of ${BhaB}_{C}^{Trp}$ (Fig. 4*B*). However, based on the CFE experiments, none of these interactions, if predicted correctly, are critical as shown by the Ala-block variants and the Ala-block insertions (Fig. 2*B*). We also confirmed the nonessentiality of these interactions with a complementary set of mutations of the enzyme. The S619D variant of ${BhaB}_{C}^{Trp}$ fully processed the WT substrate in CFE experiments as shown by MALDI-TOF MS and the S503R mutant also did not impair the activity of ${BhaB}_{C}^{Trp}$ (*SI Appendix,* Fig. S13).

Based on the AlphaFold3 model, the residues that comprise the critical PLALA C-terminal motif bend away from the preceding helical axis by about 40 degrees and turn toward the active site pocket (Fig. 4*C*). The isobutyl groups of Leu37 and Leu39 are pointing out of a groove that guides the terminus of the peptide to the active site (Fig. 4*C*). This predicted conformation provides an explanation why substitution of these Leu residues with Phe was tolerated by the enzyme, but it does not explain why experimentally the C-terminal PAAAA mutant was not a substrate for ${BhaB}_{C}^{Trp}$ (Fig. 2). AlphaFold relies on covariance in natural sequences to predict intra- and intermolecular interactions. As such, it has been shown to not be a good tool to interrogate non-natural mutations (55–58), such as those introduced in our CFE experiments. We speculate that the secondary structure of the PAAAA mutant is perturbed, possibly by extending the helix and making the 40° turn less favorable or by introducing alternative binding interactions of the terminal residues with the enzyme that similarly prevents the C terminus from turning into the active site.

${BhaB}_{C}^{Ala}$ adds an Ala residue to BhaA, a peptide that differs by only one amino acid from the substrate for ${BhaB}_{C}^{Trp}$ (Fig. 1*B*). How the two enzymes (27% sequence identity) differentiate between these two very similar peptides is not understood. We made an AlphaFold3 complex consisting of ${BhaB}_{C}^{Ala}$, tRNA^Ala^, BhaA, and ATP to investigate whether these models have enough information to explain the observed selectivity (*SI Appendix*, Fig. S15). The overall structure of the two complexes is very similar with respect to tRNA and ATP binding, but the conformations of the peptide substrates are quite different and pLDDT values are of low confidence for most of the peptide (*SI Appendix,* Fig. S15 *C* and *D*). The models provide no obvious interactions to explain the observed selectivity, possibly because the amino acids attached to the tRNA may change the observed binding poses. Further biochemical studies will be required to better understand this aspect of PEARL catalysis.

The AlphaFold3 model of BhaA-Ala binding to ${BhaB}_{C}^{Trp}$ provides a potential explanation for the observed tolerance regarding the C-terminal residue of the peptide substrate. The model suggests that there may be a pocket that is available between a partially disordered helix from Ile580 to His590 and a beta sheet from Asp30 to Ser35 that may accommodate the various side chains of the variants in Fig. 2 *B*, panel *iv* (*SI Appendix,* Fig. S16).

### LEEIEDKVAPLALA as a Tag for Trp Appendage to the C Termini of Proteins.

**Fig. 5.**
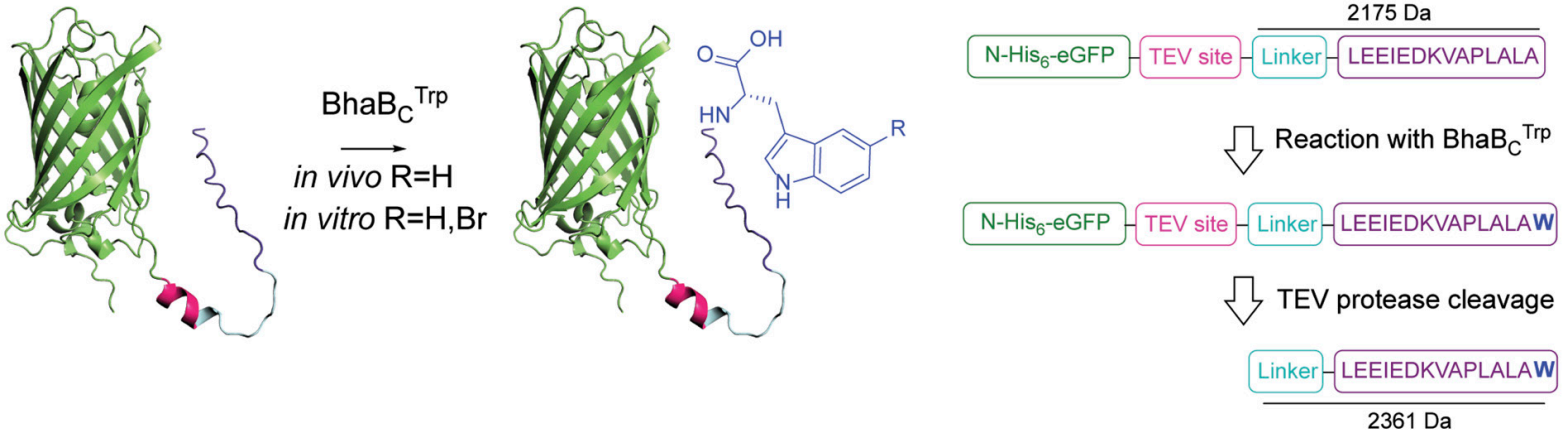
Representative example of the strategy used to append Trp or 5-Br-Trp to the C terminus of proteins, shown here for eGFP. The TEV site was added for ease of analysis by MALDI-TOF MS in *SI Appendix,* Figs. S17–S19.

The CFE evaluation of substrate specificity showed that the C-terminal 12-mer of BhaA-Ala was a substrate for ${BhaB}_{C}^{Trp}$ but with poor conversion (Fig. 2*B*). The AlphaFold3 model suggested that the 14-mer sequence of BhaA-Ala from Leu27 to Ala40 forms a helical structure (Fig. 4 *B* and *C*). Thus, we tested this 14-mer sequence (LEEIEDKAVAPLALA) as a tag to the C terminus of Enhanced Green Fluorescent Protein (eGFP), lysozyme, and maltose-binding protein (MBP) in proof-of-concept experiments to assess whether Trp could be enzymatically added to these proteins. An N-terminal His_6_-tag was added and a C-terminal TEV-cleavage site, followed by a SGSSGGSSG linker and the 14-mer BhaA-Ala sequence (e.g., Fig. 5). Each fusion protein was expressed in *E. coli* along with ${BhaB}_{C}^{Trp}$. The proteins were purified by metal affinity chromatography, treated with TEV protease, and the C-terminal peptide was analyzed by MALDI-TOF MS. A similar procedure was performed with protein substrates expressed without ${BhaB}_{C}^{Trp}$ as a control. For all three tested proteins incorporation of a C-terminal Trp was observed when coexpressed with ${BhaB}_{C}^{Trp}$ with some unmodified protein also observed (*SI Appendix,* Figs. S17–S19).

We next used purified fusion proteins for in vitro assays with purified ${BhaB}_{C}^{Trp}$, *E. coli* TrpRS, and in vitro transcribed tRNA^Trp^ and observed full incorporation of Trp (*SI Appendix,* Figs. S17–S19). Addition of proteinogenic amino acids to the C terminus of proteins has limited utility as the same amino acids could have been encoded on the encoding DNA. PEARLs or future engineered variants have the potential, however, to add noncanonical amino acids, either using substrate-tolerant natural amino acyl-tRNA synthetases or their evolved variants. As an example, we selected L-5-bromo-Trp as a noncanonical amino acid as it has previously been shown to be tolerated by *E. coli* tryptophanyl-tRNA synthetase (TrpRS) (59) and has been used for site-selective conjugation chemistry (47, 60). Indeed, 5-Br-Trp could be incorporated, albeit only to a certain extent as we also observed incorporation of nonbrominated Trp (*SI Appendix,* Fig. S20). This result was puzzling as Trp was not added to the assay and NMR characterization of 5-Br-Trp did not show contamination. Previous crystallographic studies have alluded to the possibility that Trp-AMP can copurify with TrpRS (61), thus we presumed that the observed contaminant might originate from our purified TrpRS. To solve this issue, we next used a procedure where TrpRS was incubated with excess L-5-Br-Trp and ATP to wash out any bound Trp-AMP (*SI Appendix*). Using this TrpRS for in vitro experiments allowed improved incorporation of 5-Br-Trp into the C-terminal modified protein substrates (*SI Appendix,* Figs. S17–S19, *Bottom* panels).

## Conclusion and Outlook

By utilizing a cell-free expression system, the peptide substrate requirements of the PEARL ${BhaB}_{C}^{Trp}$ were examined in a faster manner than traditional mutagenesis using a heterologous expression host for which the success rates of functional expression are still low (62). The enzyme showed unexpected tolerance to changes to the C-terminal position of the substrate BhaA-Ala and the part of the substrate that was anticipated to interact with the RRE was dispensable for enzyme activity. RREs are ubiquitous in RiPP systems, and in many cases, their interactions with the LP are well documented biochemically or crystallographically (26, 54, 63–67). However, such interactions have not yet been shown for PEARLs, and examples of vestigial RREs have been reported previously (68). In support of the general nonessentiality of the substrate segment that was expected to interact with the RRE, investigation of another PEARL, the Cys-adding TglB from *Pseudomonas syringae*, also showed that the enzyme remained active when only the C-terminal twelve residues of its substrate TglA were present (20). Instead of the expected RRE-interacting segment, the CFE data and the AlphaFold3 prediction of substrate engagement points to the importance of a C-terminal helix and the PLALA sequence that veers the C terminus into the active site. Using the information gleaned from the CFE experiments and AlphaFold3 model, a tag was developed allowing the conjugation of a Trp residue at the C terminus of three model proteins, eGFP, MBP, and lysozyme. These experiments suggest that PEARLs may have utility for protein labeling.

The enhanced abilities of AlphaFold3 to predict binding of cofactors, metal ions, and nucleotides provided a remarkable picture of a potential quaternary complex that appears to be primed for catalysis. Although the tRNA in the model is not charged with Trp and does not contain any posttranscriptional modifications, the C terminus of the peptide is right next to the γ-phosphate of ATP and the 3′-end of the tRNA is in close proximity to the C terminus of the peptide. The model explains previous site-directed mutagenesis results with TglB and was used to design mutants of ${BhaB}_{C}^{Trp}$ that were predicted to disrupt the interactions with the 3′-CCA sequence of the tRNA. In agreement with the model, the designed mutants showed strongly diminished activity. The model also suggests intriguing additional interactions with the anticodon loop that can be tested with methods that are beyond the scope of the current study. Importantly, although the model is suggestive of two sequential reactions from a quaternary complex, in the absence of kinetic studies we cannot rule out a ping-pong type mechanism in which ADP leaves the active site before the aminoacyl-tRNA enters (23).

This investigation provides a platform for future studies to test the possibility of engineering PEARLs for non-natural substrates. On the one hand, the substrate tolerance demonstrated in this work is promising. On the other hand, several questions will need to be resolved to facilitate targeted directed evolution approaches toward new activities. These include understanding the details of the sites of recognition of the tRNA sequence, and the still puzzling observation that these enzymes never add more than one residue. While this feature is beneficial in the context of peptide synthesis, it is somewhat surprising given the variety of peptides that were accepted as substrates in this study.

## Materials and Methods

Plasmids and PCR constructs were made with the primers listed in *SI Appendix*. CFE assays consisted of 5 μL reactions using the PURExpress**®** in vitro protein synthesis kit. Each reaction contained 2.5 μL of solution A, 1 μL of solution B, 0.2 μL murine RNase inhibitor, and 1.0 μL of substrate DNA solution (100 ng of template), or 1 μL of 300 to 400 ng/μL enzyme plasmid solution and 0.8 μL of 100 to 200 ng/μL substrate plasmid solution. The reactions were conducted for 5 h at 37 °C in a heating block, desalted, and analyzed by MALDI-TOF MS. Detailed procedures for CFE, protein purification, and MS are provided in *SI Appendix*. For the protein tagging experiments, pACYCDuet-1 plasmids encoding tagged eGFP, lysozyme, and MBP were cotransformed with a pET28a plasmid encoding ${BhaB}_{C}^{Trp}$. After Ni-NTA purification, the eluted His-tagged protein substrates were digested with 1 µg tobacco etch virus (TEV) in 50 mM Tris-HCl pH 8 and 1 mM DTT overnight at 4 °C followed by MS analysis. All primary data for all figures have been deposited at Mendeley data (https://data.mendeley.com/datasets/88tzntsmhs/1) (69).

## Supplementary Material

Appendix 01 (PDF)

Dataset S01 (PDB)

## Data Availability

Mass spectrometry data have been deposited in Mendeley data (https://doi.org/10.17632/88tzntsmhs.1) (69). All other data are included in the manuscript and/or supporting information.
