# Supplementary material for: Substrate recognition by a peptide-aminoacyl-tRNA ligase
Source: Proc Natl Acad Sci U S A. 2025 Mar 19;122(12):e2423858122. doi: 10.1073/pnas.2423858122 (PMC11962472; doi:10.1073/pnas.2423858122)
Supplement: Supplementary file 1 — Appendix 01 (PDF) [file pnas.2423858122.sapp.pdf]

Supplementary Information Appendix

Substrate recognition by a peptide-aminoacyl-tRNA ligase

Josseline Ramos Figueroa,[1,#] Haoqian Liang,[2,#] and Wilfred A. van der Donk[1,2,*]

[1] Department of Chemistry and Howard Hughes Medical Institute, University of Illinois at Urbana-Champaign, Urbana, Illinois 61801, USA.

[2] Department of Biochemistry University of Illinois at Urbana-Champaign, Urbana, Illinois 61801, USA.

*vddonk@illinois.edu

[#] These authors contributed equally to this study

Primary data associated with this study have been deposited in:

Ramos Figueroa, Josseline; Liang, Lainey (Haoqian); van der Donk, Wilfred (2025), "Data associated with "Substrate recognition by a peptide-aminoacyl-tRNA ligase"", Mendeley Data, V1, doi: 10.17632/88tzntsmhs.1

Materials and Methods

Primers and gBlocks were ordered from Integrated DNA Technologies. Q5 HF DNA Polymerase, PURExpress® *in vitro* protein synthesis kit and murine RNase inhibitor were obtained from New England Biolabs.(1) Super DHB for use as matrix in MALDI-TOF MS was purchased from Sigma-Aldrich. C_{18} resin ziptip pipette tips were purchased from Millipore Sigma. The NuPAGE™ 4%-12%, Bis-Tris, precast polyacrylamide gels, NuPAGE™ LDS sample buffer (4X), NuPAGE™ MES SDS running buffer (20X), and NuPAGE™ sample reducing agent (X10) used for SDS-

PAGE were purchased from ThermoFisher. Precision Plus Protein™ All Blue Protein ladder was purchased from Bio-Rad. Molecular grade water, and RNase/DNase free low-binding tubes were purchased from Fisher Scientific. DNA miniprep kits were purchased from Qiagen. Polymerase chain reaction (PCR) was performed using a Bio-Rad C1000 thermocycler. MALDI-TOF MS analysis was carried out in the mass spectrometry facility at UIUC using a Bruker Daltonics UltrafleXtreme MALDI-TOF mass spectrometer. MALDI-TOF MS data were calibrated using Protein Calibration Standard I purchased from Bruker and processed using the software FlexAnalysis. Structural predictions were performed using the AlphaFold3 server.

Cloning, plasmid construction, and expression

All primers and gBlocks used for cloning, sequencing and CFE DNA templates are listed in Table S1. Plasmids containing genes from the *bha* BGC including pET28a-His_6-BhaA-Ala (MCSI), pET28a-His_6–$BhaB_7$ (MCSI) (renamed to $BhaB_C^{Trp}$ based on its characterized function (2)), and pET28a-His_6-EcTrpRS (containing *E. coli* tryptophan-tRNA ligase, TrpRS) were cloned as previously described (2). Notably, both genes were not *E. coli* codon-optimized and maintained the native sequences as obtained from the *bha* BGC genomic data. *E. coli* Dh10β was used as the host for plasmid propagation. The double-stranded DNA fragment containing synthetic DNA encoding BhaA-Ala variants was designed as shown in Figure S2. Plasmids containing eGFP, lysozyme, and MBP constructs as shown in Figure S13 were amplified from the corresponding gBlocks (Table S1) and inserted into pACYCDuet-1 vectors using Gibson Assembly. NEB-10beta competent *E. coli* cells were used for transformation and plasmids isolated from single colonies were submitted to sequencing. Successful plasmid constructs were then used in expression experiments. Plasmids containing single-point $BhaB_C^{Trp}$ mutants were constructed by a two-piece

Gibson Assembly using ~300bp-long gBlocks containing the desired single-point mutation and the amplified PCR product from pET28a-His_6–$BhaB_7$ (MCSI) bearing the corresponding overlapping regions. Modified eGFP, lysozyme, and MBP fusion proteins, *E. coli* TrpRS, as well as the wild type $BhaB_C^{Trp}$ and its variants were all individually transformed into *E. coli* BL21 (DE3) electrocompetent cells. Single colonies were used to inoculate 1 to 3 L LB cultures with the corresponding antibiotics and grown overnight at 37 °C and shaken at 200 RPM for 3 h or until OD600 reached 0.6-0.8 units. After inoculation with IPTG to 0.4 mM final concentration, the cultures were further grown overnight at 18 °C and shaken at 200 RPM. After that, cells were harvested in a centrifugal device at 5,200 ×*g* and the pellets were resuspended in native buffer (50 mM HEPES, 300 mM NaCl, and 50 mM imidazole, 5% glycerol at pH 7.5) containing Pierce protein inhibitor cocktail and 1 mM TCEP. Next, modified eGFP, lysozyme, and MBP fusion protein containing resuspensions were processed by sonication with cycles of 2 s on and 5 s off. TrpRS and $BhaB_C^{Trp}$ were instead homogenized using an Avestin EmulsiFlex C3 equipment at 15000 psi requiring five to seven passes. After cell lysis, centrifugation was performed at 50,000 ×*g* for 30 min and the supernatant was loaded onto a Ni-NTA resin previously equilibrated with the start buffer. After washing with 20 CV of the same initial buffer to remove non-specific protein binding, the His-tagged proteins were eluted with 5 CV of 50 mM HEPES, 300 mM NaCl, 200 mM imidazole, 5% glycerol at pH 7.5. The excess imidazole content was then removed by concentration and buffer exchange into 50 mM HEPES, 150 mM NaCl, 5% glycerol at pH 7.5 using the appropriate MWCO Amicon centrifugal device.

CFE assay

First, pET28a-His$_6$-BhaA-Ala and pET28a-His$_6$–BhaB$_7$ plasmids were used to transform *E. coli* Dh10β cells and a single colony was used for amplification in 10 mL of Luria-Bertani medium (LB) containing 100 µg/mL of kanamycin. After overnight culture, the cells were spun down at 4,500 rpm for 10 min at 25 °C. Plasmid DNA was then extracted from the cell pellet by using a miniprep kit (RNase added) and re-dissolved in molecular grade water. Synthetic double-stranded DNA fragments of BhaA-Ala mutants were dissolved in molecular grade water and applied in CFE directly without additional purification.

CFE assays usually consisted of 5 µL reactions using the PURExpress® *in vitro* protein synthesis kit. Each reaction contained 2.5 µL of solution A, 1 µL of solution B and 0.2 µL murine RNase inhibitor, and 1.0 µL of substrate solution (containing 100 ng of template) or 1.8 µL total volume of both substrate and enzyme (containing 1 µL of 300-400 ng/µL enzyme plasmid solution and 0.8 µL of 100-200 ng/µL substrate plasmid solution). All reagents were usually thawed over ice. The reactions were set up in pre-chilled 200-µL tubes, and all reagents were added in a specific order as suggested by the manufacturer. Solution A was added first, then solution B, and last the RNase inhibitor followed by DNA templates. For the reactions that contained enzyme and substrate, the DNA template for the enzyme was added first. The reaction was conducted for 5 h at 37 °C in a heating block, desalted, and analyzed by MALDI-TOF MS. Rough estimation of conversion was determined by the intensity of the peaks for starting material and product observed in two independent experiments. Peptide sequence can strongly influence MALDI-TOF MS intensities, but in this investigation the sequences of the peptides are almost identical between substrate and product, differing only in the addition of a C-terminal Trp. CFE does not result in sufficient amounts of material for LC-MS analysis.

Additionally, SDS-PAGE was used to visualize enzyme expression. Typically, the 5 μL reaction containing either enzyme plasmid alone or plasmids for both substrate and enzyme was combined with 4 μL of sample buffer, 1.5 μL of reducing agent, and water to a final volume of 15 μL. The sample was heated at 70 ºC for 5 min, then loaded in the gel. The gel was run for 20 min at 200 V in freshly prepared 1x MES SDS buffer, and the protein bands were visualized by Coomassie staining. A control 5 μL reaction containing no DNA template was used. $BhaB_C^{Trp}$ mutants were similarly evaluated using plasmid encoding BhaA-Ala and following the CFE protocol described in this section.

Use of $BhaB_C^{Trp}$ *in vivo* and *in vitro* for Trp or Br-Trp addition to proteins

For the *in vivo* experiment, pACYCDuet-1 plasmids bearing modified eGFP, lysozyme, and MBP sequences were first co-transformed with plasmid bearing $BhaB_C^{Trp}$ (pET28a-His_6–$BhaB_7$ (MCSI)) using electrocompetent BL21 (DE3). Single colonies were then taken and used as inoculants for the following expression protocols as described earlier. After that, cells were lysed and processed as indicated earlier. After Ni-NTA purification, the eluted His-tagged protein substrates were buffer exchanged, and their concentrations were roughly estimated as his_6 tagged $BhaB_C^{Trp}$ was also present in the elution. Next, 10-20 μM recovered protein was digested with 1 μg tobacco etch virus (TEV) in a buffer containing 50 mM Tris-HCl pH 8 and 1 mM DTT overnight at 4 °C. A similar procedure was performed to digest pure eGFP, lysozyme, and MBP fusion constructs obtained in the absence of $BhaB_C^{Trp}$ as control samples. Last, after TEV digestion, samples were desalted and submitted for MALDI-TOF MS analysis to evaluate the incorporation of Trp into the C-terminal of the protein substrates.

For *in vitro* experiments, previously described protocols were followed (3). Briefly, a final concentration of 10-30 μM protein substrate, 10 μM $BhaB_C^{Trp}$, 50 μM TrpRS, 1 μg of in vitro transcribed $tRNA^{Trp}$ (prepared using HiScribe T7 High Yield RNA Synthesis kit as described in the previous protocol), 10 U Superase, 30 U Thermostable Inorganic Pyrophosphatase (TIPP), 2 mM DTT, 5 mM Trp or L-5-Br-Trp, and 6 mM ATP were combined in a buffer such that the final concentration was 50 mM HEPES, 25 mM KCl, and 15 mM $MgCl_2$ pH 7.6. The reaction was incubated at 37 °C for 4 h in a heating block. After this time, 25 μL aliquot was taken for TEV digestion using 2 μg of enzyme in 1 mM DTT, 50 mM Tris-HCl pH 8 buffer set overnight at 4 °C. Last, the digested samples were desalted and submitted for MALDI-TOF MS analysis, a reaction without addition of TrpRS and $tRNA^{Trp}$ was used as control.

For the reactions using L-5-Br-Trp, a wash procedure was implemented to remove Trp-AMP that has been reported to copurify with *E. coli* TrpRS (4). A 500-μL aliquot containing 50 mM HEPES, 300 mM NaCl, pH 7.5, 5% glycerol, 1 mM L-5-Br-Trp, and 1 mM ATP was added to a 50-μL aliquot of purified TrpRS, incubated at room temperature for 10 min, and centrifuged through a 30 kDa molecular weight cutoff membrane at 10,000 ×g for 5 min. The process was repeated four times. The resulting TrpRS aliquots were used for *in vitro* studies using $BhaB_C^{Trp}$ and enzyme fusion constructs for Br-Trp addition (bottom panels of Figures S17-S19).

Additional discussion of BhaA-Ala binding to $BhaB_C^{Trp}$

As noted in the main text, the prediction of the conformation of the N-terminus of BhaA-Ala bound to $BhaB_C^{Trp}$ has significantly lower pLDDT values than the C-terminus and we are less confident about the accuracy of the predictions. We will first describe the model and then discuss it in terms of experimental data. Only three polar interactions are observed between the N-terminal helical

structure and $BhaB_C^{Trp}$, with Glu9 and Thr6 of BhaA-Ala predicted to hydrogen bond with Lys332, Asp19 to interact with Lys293, and Asp21 to engage Lys339 (Figure S21A). While these observations are consistent with the highly conserved presence of these residues in related peptides, the Ala-block scanning mutagenesis suggests that the polar interactions with these two helical segments might be dispensable as Ala substitutions did not abolish $BhaB_C^{Trp}$ activity. Furthermore, the predicted conformation suggests that the N-terminus of BhaA-Ala does not interact with the RiPP recognition element (RRE) that is present in $BhaB_C^{Trp}$. Many RiPP biosynthetic enzymes have been shown to engage with their substrates using RREs (5-7), with the interaction usually involving a segment of the substrate attaining a β-strand conformation that complements a four-stranded β-sheet in the RRE, forming an antiparallel arrangement (e.g. Figure S21B and Figure S21D left panel, for NisB (8, 9)). AlphaFold3 does not predict such an interaction even though the RRE of $BhaB_C^{Trp}$ is close to the N-terminal helix of BhaA-Ala in the model (Figure S21C and Figure S21D right panel). Given the lower pLDDT values, one could envision BhaA-Ala attaining a different conformation in which the N-terminus would engage with the RRE. Future studies will be needed to confirm or refute the AlphaFold3 prediction, but as noted this part of the substrate is not required for activity and hence regardless of the accuracy of the prediction, we do not believe it affects the conclusions regarding the important features of substrate recognition. We note reports of other experimentally confirmed examples of substrate recognition in RRE containing enzymes that do not engage using the canonical β-sheet mechanism (10).

Table S1. List of primers and double stranded DNA fragments applied in this study.

DNA Name	Sequence
LL_BhaAA_Truncate_1_noHis	GGCGTAATACGACTCACTATAGGGTTAACTTTAACAAGGAGAAAAACATGATCGACTTTGATCTAGAGGAAATTGAAGATAAAGTCGCTCCTCTGGCTCTTGCGTAATAAGCTTCGCGGTTCGAAGCTA
LL_BhaAA_Truncate_2_noHis	GGCGTAATACGACTCACTATAGGGTTAACTTTAACAAGGAGAAAAACATGGAAATTGAAGATAAAGTCGCTCCTCTGGCTCTTGCGTAATAAGCTTCGCGGTTCGAAGCTAGCATAACCCCTTGGGGCC
LL_BhaAA_Truncate_3	GGCGTAATACGACTCACTATAGGGTTAACTTTAACAAGGAGAAAAACATGGCCGACAAGGTTACACCTGAAGAAGAGTTAGATCTAGAATTAGAAATTGAAGACCTTGATGACATCGACTTTGATCTAGAGGAAATTCCTCTGGCTCTTGCGTAAGCTTCG
LL_BhaAA(FDLE_AAAA)_noHis	GGCGTAATACGACTCACTATAGGGTTAACTTTAACAAGGAGAAAAACATGGCCGACAAGGTTACACCTGAAGAAGAGTTAGATCTAGAATTAGAAATTGAAGACCTTGATGACATCGACGCGGCGGCGGCGGAAATTGAAGATAAAGTCGCTCCTCTGGCTCTTGCGTAAGCTTCG
LL_BhaAA(LEEI_AAAA)_noHis	GGCGTAATACGACTCACTATAGGGTTAACTTTAACAAGGAGAAAAACATGGCCGACAAGGTTACACCTGAAGAAGAGTTAGATCTAGAATTAGAAATTGAAGACCTTGATGACATCGACTTTGATGCGGCGGCGGCGGAAGATAAAGTCGCTCCTCTGGCTCTTGCGTAAGCTTCG
LL_BhaAA(EIED_AAAA)_noHis	GGCGTAATACGACTCACTATAGGGTTAACTTTAACAAGGAGAAAAACATGGCCGACAAGGTTACACCTGAAGAAGAGTTAGATCTAGAATTAGAAATTGAAGACCTTGATGACATCGACTTTGATCTAGAGGCGGCGGCGGCGAAAGTCGCTCCTCTGGCTCTTGCGTAAGCTTCG
LL_BhaAA(EDKV_AAAA)_noHis	GGCGTAATACGACTCACTATAGGGTTAACTTTAACAAGGAGAAAAACATGGCCGACAAGGTTACACCTGAAGAAGAGTTAGATCTAGAATTAGAAATTGAAGACCTTGATGACATCGACTTTGATCTAGAGGAAATTGCGGCGGCGGCGGCTCCTCTGGCTCTTGCGTAAGCTTCG
LL_BhaAA(KVAP_AAAA)_noHis	GGCGTAATACGACTCACTATAGGGTTAACTTTAACAAGGAGAAAAACATGGCCGACAAGGTTACACCTGAAGAAGAGTTAGATCTAGAATTAGAAATTGAAGACCTTGATGACATCGACTTTGATCTAGAGGAAATTGAAGATGCGGCGGCGGCGCTGGCTCTTGCGTAAGCTTCG
LL_BhaAA(LALA_AAAA)_noHis	GGCGTAATACGACTCACTATAGGGTTAACTTTAACAAGGAGAAAAACATGGCCGACAAGGTTACACCTGAAGAAGAGTTAGATCTAGAATTAGAAATTGAAGACCTTGATGACATCGACTTTGATCTAGAGGAAATTGAAGATAAAGTCGCTCCTGCGGCGGCGGCGTAAGCTTCG

LL_BhaAA_APLA_AALA	GGCGTAATACGACTCACTATAGGGTTAACTTTAACAAGGAGAAAAACATGGCCGACAAGGTTACACCTGAAGAAGAGTTAGATCTAGAATTAGAAATTGAAGACCTTGATGACATCGACTTTGATCTAGAGGAAATTGAAGATAAAGTCGCTGCTCTGGCTCTTGCTTAAGCTTCG
LL_BhaAA_LALA_LAAA	GGCGTAATACGACTCACTATAGGGTTAACTTTAACAAGGAGAAAAACATGGCCGACAAGGTTACACCTGAAGAAGAGTTAGATCTAGAATTAGAAATTGAAGACCTTGATGACATCGACTTTGATCTAGAGGAAATTGAAGATAAAGTCGCTCCTCTTGCGGCCGCGTAAGCTTCG
LL_BhaAA_LALA_AALA	GGCGTAATACGACTCACTATAGGGTTAACTTTAACAAGGAGAAAAACATGGCCGACAAGGTTACACCTGAAGAAGAGTTAGATCTAGAATTAGAAATTGAAGACCTTGATGACATCGACTTTGATCTAGAGGAAATTGAAGATAAAGTCGCTCCTGCAGCGCTTGCGTAAGCTTCG
LL_BhaAA_LALA_FALA	GGCGTAATACGACTCACTATAGGGTTAACTTTAACAAGGAGAAAAACATGGCCGACAAGGTTACACCTGAAGAAGAGTTAGATCTAGAATTAGAAATTGAAGACCTTGATGACATCGACTTTGATCTAGAGGAAATTGAAGATAAAGTCGCTCCTTTTGCGCTTGCGTAAGCTTCG
LL_BhaAA_LALA_LAFA	GGCGTAATACGACTCACTATAGGGTTAACTTTAACAAGGAGAAAAACATGGCCGACAAGGTTACACCTGAAGAAGAGTTAGATCTAGAATTAGAAATTGAAGACCTTGATGACATCGACTTTGATCTAGAGGAAATTGAAGATAAAGTCGCTCCTCTTGCGTTTGCGTAAGCTTCG
LL_BhaAA_LALA_LFLA	GGCGTAATACGACTCACTATAGGGTTAACTTTAACAAGGAGAAAAACATGGCCGACAAGGTTACACCTGAAGAAGAGTTAGATCTAGAATTAGAAATTGAAGACCTTGATGACATCGACTTTGATCTAGAGGAAATTGAAGATAAAGTCGCTCCTCTTTTTCTTGCGTAAGCTTCG
LL_BhaAA_L	GGCGTAATACGACTCACTATAGGGTTAACTTTAACAAGGAGAAAAACATGGCCGACAAGGTTACACCTGAAGAAGAGTTAGATCTAGAATTAGAAATTGAAGACCTTGATGACATCGACTTTGATCTAGAGGAAATTGAAGATAAAGTCGCTCCTCTGGCTCTTCTGTAAGCTTCG
LL_BhaAA_G	GGCGTAATACGACTCACTATAGGGTTAACTTTAACAAGGAGAAAAACATGGCCGACAAGGTTACACCTGAAGAAGAGTTAGATCTAGAATTAGAAATTGAAGACCTTGATGACATCGACTTTGATCTAGAGGAAATTGAAGATAAAGTCGCTCCTCTGGCTCTTGGCTAAGCTTCG
LL_BhaAA_F	GGCGTAATACGACTCACTATAGGGTTAACTTTAACAAGGAGAAAAACATGGCCGACAAGGTTACACCTGAAGAAGAGTTAGATCTAGAATTAGAAATTGAAGACCTTGATGACATCGACTTTGATCTAGAGGAAATTGAAGATAAAGTCGCTCCTCTGGCTCTTTTTTAAGCTTCG

LL_BhaAA_P	GGCGTAATACGACTCACTATAGGGTTAACTTTAACAAGGAGAAAAACATGGCCGACAAGGTTACACCTGAAGAAGAGTTAGATCTAGAATTAGAAATTGAAGACCTTGATGACATCGACTTTGATCTAGAGGAAATTGAAGATAAAGTCGCTCCTCTGGCTCTTCCTTAAGCTTCG
LL_BhaAA_S	GGCGTAATACGACTCACTATAGGGTTAACTTTAACAAGGAGAAAAACATGGCCGACAAGGTTACACCTGAAGAAGAGTTAGATCTAGAATTAGAAATTGAAGACCTTGATGACATCGACTTTGATCTAGAGGAAATTGAAGATAAAGTCGCTCCTCTGGCTCTTTCTTAAGCTTCG
LL_BhaAA_K	GGCGTAATACGACTCACTATAGGGTTAACTTTAACAAGGAGAAAAACATGGCCGACAAGGTTACACCTGAAGAAGAGTTAGATCTAGAATTAGAAATTGAAGACCTTGATGACATCGACTTTGATCTAGAGGAAATTGAAGATAAAGTCGCTCCTCTGGCTCTTAAATAAGCTTCG
LL_BhaAA_E	GGCGTAATACGACTCACTATAGGGTTAACTTTAACAAGGAGAAAAACATGGCCGACAAGGTTACACCTGAAGAAGAGTTAGATCTAGAATTAGAAATTGAAGACCTTGATGACATCGACTTTGATCTAGAGGAAATTGAAGATAAAGTCGCTCCTCTGGCTCTTGAATAAGCTTCG
LL_BhaAA_W	GGCGTAATACGACTCACTATAGGGTTAACTTTAACAAGGAGAAAAACATGGCCGACAAGGTTACACCTGAAGAAGAGTTAGATCTAGAATTAGAAATTGAAGACCTTGATGACATCGACTTTGATCTAGAGGAAATTGAAGATAAAGTCGCTCCTCTGGCTCTTTGGTAAGCTTCG
LL_BhaAA_Y	GGCGTAATACGACTCACTATAGGGTTAACTTTAACAAGGAGAAAAACATGGCCGACAAGGTTACACCTGAAGAAGAGTTAGATCTAGAATTAGAAATTGAAGACCTTGATGACATCGACTTTGATCTAGAGGAAATTGAAGATAAAGTCGCTCCTCTGGCTCTTTATTAAGCTTCG
LL_BhaAA_Elongate	GGCGTAATACGACTCACTATAGGGTTAACTTTAACAAGGAGAAAAACATGGCCGACAAGGTTACACCTGAAGAAGAGTTAGATCTAGAATTAGAAATTGAAGACCTTGATGACATCGACTTTGATCTAGAGGAAATTGAAGATAAAGTCGCGGCGGCTGCGGCCGCTCCTCTGGCGCTTGCGTAAGCTTCG
LL_BhaAA_Elongate2	GGCGTAATACGACTCACTATAGGGTTAACTTTAACAAGGAGAAAAACATGGCCGACAAGGTTACACCTGAAGAAGAGTTAGATCTAGAATTAGAAATTGAAGACCTTGATGACATCGACTTTGATCTAGAGGAAATTGAAGATAAAGTCGCGGCGGCTGCGGCCAAAGTCGCTCCTCTGGCTCTTGCGTAATAAGCTTCG
LL_BhaAA_Elongate3	GGCGTAATACGACTCACTATAGGGTTAACTTTAACAAGGAGAAAAACATGGCCGACAAGGTTACACCTGAAGAAGAGTTAGATCTAGAATTAGAAATTGAAGACCTTGATGACATCGACTTTGATCTAGAGGAAATTGAAGATAAAGTCGC

	GGCGGCTGCGGCCGAAGATAAAGTCGCTCCTCTGGCTC<br>TTGCGTAATAAGCTTCG
LL_eGFP_LEEIEDKV_C<br>T	GGCGTAATACGACTCACTATAGGGTTAACTTTAACAAG<br>GAGAAAAACATGGGCAGCAGCCATCACCATCATCACC<br>ACAGCAGCGGCAGCAGCGGCCTGGTGCCGCGCGGCAG<br>CATGGCTAGCATGGTGAGCAAGGGCGAGGAGCTGTTC<br>ACCGGGGTGGTGCCCATCCTGGTCGAGCTGGACGGCG<br>ACGTAAACGGCCACAAGTTCAGCGTGTCCGGCGAGGG<br>CGAGGGCGATGCCACCTACGGCAAGCTGACCCTGAAG<br>TTCATCTGCACCACCGGCAAGCTGCCCGTGCCCTGGCC<br>CACCCTCGTGACCACCCTGACCTACGGCGTGCAGTGCT<br>TCAGCCGCTACCCCGACCACATGAAGCAGCACGACTTC<br>TTCAAGTCCGCCATGCCCGAAGGCTACGTCCAGGAGCG<br>CACCATCTTCTTCAAGGACGACGGCAACTACAAGACCC<br>GCGCCGAGGTGAAGTTCGAGGGCGACACCCTGGTGAA<br>CCGCATCGAGCTGAAGGGCATCGACTTCAAGGAGGAC<br>GGCAACATCCTGGGGCACAAGCTGGAGTACAACTACA<br>ACAGCCACAACGTCTATATCATGGCCGACAAGCAGAA<br>GAACGGCATCAAGGTGAACTTCAAGATCCGCCACAAC<br>ATCGAGGACGGCAGCGTGCAGCTCGCCGACCACTACC<br>AGCAGAACACCCCCATCGGCGACGGCCCCGTGCTGCT<br>GCCCGACAACCACTACCTGAGCACCCAGTCCGCCCTGA<br>GCAAAGACCCCAACGAGAAGCGCGATCACATGGTCCT<br>GCTGGAGTTCGTGACCGCCGCCGGGATCACTCTCGGCG<br>GCTCTAGCGGTGGCTCCAGCGGTCTAGAGGAAATTGA<br>AGATAAAGTCGCTCCTCTGGCTCTTGCGTAATAAGCTT<br>CG
LL_Lysozyme_LEEIEDK<br>V_CT	GGCGTAATACGACTCACTATAGGGTTAACTTTAACAAG<br>GAGAAAAACATGGGCAGCAGCCATCACCATCATCACC<br>ACAGCAGCGGCAGCAGCGGCCTGGTGCCGCGCGGCAG<br>CATGAATATATTTGAAATGTTACGTATAGATGAAGGTC<br>TTAGACTTAAAATCTATAAAGACACAGAAGGCTATTAC<br>ACTATTGGCATCGGTCATTTGCTTACAAAAAGTCCATC<br>ACTTAATGCTGCTAAATCTGAATTAGATAAAGCTATTG<br>GGCGTAATTGCAATGGTGTAATTACAAAAGATGAGGC<br>TGAAAAACTCTTTAATCAGGATGTTGATGCTGCTGTTC<br>GCGGAATTCTGAGAAATGCTAAATTAAAACCGGTTTAT<br>GATTCTCTTGATGCGGTTCGTCGCTGTGCATTGATTAAT<br>ATGGTTTTCCAAATGGGAGAAACCGGTGTGGCAGGATT<br>TACTAACTCTTTACGTATGCTTCAACAAAAACGCTGGG<br>ATGAAGCAGCAGTTAACTTAGCTAAAAGTAGATGGTA<br>TAATCAAACACCTAATCGCGCAAAACGAGTCATTACA<br>ACGTTTAGAACTGGCACTTGGGACGCGTATAAAAATCT<br>AGAAAACCTGTATTTTCAGAGCGGCTCTAGCGGTGGCT<br>CCAGCGGTCTAGAGGAAATTGAAGATAAAGTCGCTCC<br>TCTGGCTCTTGCGTAATAAGCTTCG

LL_MBP_LEEIEDKV_CT	GGCGTAATACGACTCACTATAGGGTTAACTTTAACAAG<br>GAGAAAAACATGGGCAGCAGCCATCACCATCATCACC<br>ACAGCAGCGGCAGCAGCGGCCTGGTGCCGCGCGGCAG<br>CATGAAAATCGAAGAAGGTAAACTGGTAATCTGGATT<br>AACGGCGATAAAGGCTATAACGGTCTCGCTGAAGTCG<br>GTAAGAAATTCGAGAAAGATACCGGAATTAAAGTCAC<br>CGTTGAGCATCCGGATAAACTGGAAGAGAAATTCCCA<br>CAGGTTGCGGCAACTGGCGATGGCCCTGACATTATCTT<br>CTGGGCACACGACCGCTTTGGTGGCTACGCTCAATCTG<br>GCCTGTTGGCTGAAATCACCCCGGACAAAGCGTTCCAG<br>GACAAGCTGTATCCGTTTACCTGGGATGCCGTACGTTA<br>CAACGGCAAGCTGATTGCTTACCCGATCGCTGTTGAAG<br>CGTTATCGCTGATTTATAACAAAGATCTGCTGCCGAAC<br>CCGCCAAAAACCTGGGAAGAGATCCCGGCGCTGGATA<br>AAGAACTGAAAGCGAAAGGTAAGAGCGCGCTGATGTT<br>CAACCTGCAAGAACCGTACTTCACCTGGCCGCTGATTG<br>CTGCTGACGGGGGTTATGCGTTCAAGTATGAAAACGGC<br>AAGTACGACATTAAAGACGTGGGCGTGGATAACGCTG<br>GCGCGAAAGCGGGTCTGACCTTCCTGGTTGACCTGATT<br>AAAAACAAACACATGAATGCAGACACCGATTACTCCA<br>TCGCAGAAGCTGCCTTTAATAAAGGCGAAACAGCGAT<br>GACCATCAACGGCCCGTGGGCATGGTCCAACATCGAC<br>ACCAGCAAAGTGAATTATGGTGTAACGGTACTGCCGA<br>CCTTCAAGGGTCAACCATCCAAACCGTTCGTTGGCGTG<br>CTGAGCGCAGGTATTAACGCCGCCAGTCCGAACAAAG<br>AGCTGGCGAAAGAGTTCCTCGAAAACTATCTGCTGACT<br>GATGAAGGTCTGGAAGCGGTTAATAAAGACAAACCGC<br>TGGGTGCCGTAGCGCTGAAGTCTTACGAGGAAGAGTT<br>GGCGAAAGATCCACGTATTGCCGCCACCATGGAAAAC<br>GCCCAGAAAGGTGAAATCATGCCGAACATCCCGCAGA<br>TGTCCGCTTTCTGGTATGCCGTGCGTACTGCGGTGATC<br>AACGCCGCCAGCGGTCGTCAGACTGTCGATGAAGCCCT<br>GAAAGACGCGCAGACTAATGGCTCTAGCGGTGGCTCC<br>AGCGGTCTAGAGGAAATTGAAGATAAAGTCGCTCCTCT<br>GGCTCTTGCGTAATAAGCTTCG
Gene_for_pACYC.F	CAGCAGCGGCCTGG
Gene_for_pACYC.R	CCCAAGGGGTTATGCTAGTTATTGCTCATTACGCAAGA<br>GCCAGAGGAGCGACTTTATCTT
BB_pACYC_for_insert.F	TTCATGCTGCCGCGCGGCACCAGGCCGCTGCTGCCGCT<br>GCTGTGGTGATGATGGTGATGG
BB_pACYC_for_insert.R	CTCCTCTGGCTCTTGCGTAATGAGCAATAACTAGCATA<br>ACCCCTTGGGGCCTCTAAACGG
BhaB7_R208A_gb	GCGGAAAAGCAATTATTCCTACAGGAGATTTATAAAA<br>ATCCCCGATTGCAGGAAGCCATTTTTCAACAAAGTCCT<br>TCAATGTATAAAAACGCAGTTGTCCCTTATGTTCACTC<br>TTCTTTACAAAAACGGAACACAAATATTAAGCGAATTG

	AACGACAGTTAATTTCCTATTTACAAGCGCTGTGTACA AAAAATGAAACCACGAGCTTCTTTGGTCCTATTCAGTA TGGAGTGTTAACCTCAGAACAACAAGACATTGAATAT AATTTCAATCAGAAGGAAACTGAACGGAGAGCTTTTAT GCCTTATTGGTCAATCAAAGTATTAGCAGCACAAATGA AAGAATGTGACGTATTC
BhaB7_Y205A_gb	GCGGAAAAGCAATTATTCCTACAGGAGATTTATAAAA ATCCCCGATTGCAGGAAGCCATTTTTCAACAAGTCCT TCAATGTATAAAAACGCAGTTGTCCCTTATGTTCACTC TTCTTTACAAAAACGGAACACAAATATTAAGCGAATTG AACGACAGTTAATTTCCGCGTTACAAAGACTGTGTACA AAAAATGAAACCACGAGCTTCTTTGGTCCTATTCAGTA TGGAGTGTTAACCTCAGAACAACAAGACATTGAATAT AATTTCAATCAGAAGGAAACTGAACGGAGAGCTTTTAT GCCTTATTGGTCAATCAAAGTATTAGCAGCACAAATGA AAGAATGTGAC
BhaB7_R429A_gb	CCTATCACTATGGCAAGAAAGATTGGAATGGCTTTGGA AAGTCAAACAAGCTTATCCTTACATGACGCTTGGGCAG AAACAAGAGCTATTTCACAAATTGGAGTCAACTTTTAC TGAGTGGACAGGTGAACAGCCTAGGCGTTTAGGCGGG GAGATTTATGCAGATGCTAATCTTTTGTATGAAGAGTG TCATGGACCGTTAAACAATATTAAAATTGGTGGGTCCA TTAAGCACGTTCTTAAACAAGATGTACCTAAGTGGCTA TCGATTTGCGCTAAACACGGGGAAC
BhaB7_S618D_gb	CCTATCAAGTCATTTTAGGCGAAATACATGACACGATT ATGGTTTGGGGATGGGCCTTACAATTTCATCCAGAAAA AGACAGAGTGAATGAACAATTAGTAAAGAAAATACAA AAAAGTACGCAACACCTTCGAATGCTTAACATGCTCGA TAGCAAACGCTTTAAAATTGTACCTTTTGAATATCCAG GTACAACGATCCAAATGAACAGCTTTTCAAACAGCCCA AATGAAAAAATTCCGTTAAGTCAATTAAAAGTAACCTA CACGAAAGAGGGGCTAGCGCTTACACTTCCTGATCATG AAGAGGTTTTTTATAC
BhaB7_S619D_gb	CAAGTCATTTTAGGCGAAATACATGACACGATTATGGT TTGGGGATGGGCCTTACAATTTCATCCAGAAAAAGACA GAGTGAATGAACAATTAGTAAAGAAAATACAAAAAAG TACGCAACACCTTCGAATGCTTAACATGCTCTCAGATA AACGCTTTAAAATTGTACCTTTTGAATATCCAGGTACA ACGATCCAAATGAACAGCTTTTCAAACAGCCCAAATG AAAAAATTCCGTTAAGTCAATTAAAAGTAACCTACACG AAAGAGGGGCTAGCGCTTACACTTCCTGATCATGAAG AGGTTTTTTATACG
BhaB7_S503R_gb	CATTAAGCACGTTCTTAAACAAGATGTACCTAAGTGGC TATCGATTTGCGCTAAACACGGGGAACAGAGGCGGAA ACAAGAACAAGCATTGGCTCAAGAGATTTTTAAAATG ATGTATCCAAATGAAGATTCTGTACCATTTCTGAAATT

	CGTTCACGACCTTAGAAATCACCCAGATGTACATACGT GGGAAAAGCGATGGCAATCAATAAAAACAGAAATAGA GGAGGCCATTACCCATGAAGTAGCGAGTAATCCTAAA TCAGTTGTCCATCTTTCTTTAGATTACCAAGACTTTGAT CGTGACATGGCATGGTTAACTTCTCCTGATCTCATGAT AGCTAAAAAAGATGATGATTCCTATCAAGTC
BhaB7_S503R_F	GTGACATGGCATGGTTAACTTCTCC
BhaB7_S503R_R	GCGCAAATCGATAGCCACTTAGG
BhaB7_S618D_F	CCTACACGAAAGAGGGGCTAGC
BhaB7_S618D_R	CATCCCCAAACCATAATCGTGTCATG
BhaB7_R429A_F	GTGGGTCCATTAAGCACGTTCTTAAAC
BhaB7_R429A_R	CAATTTGTGAAATAGCTCTTGTTTCTGCCC
BhaB7_R208A_F	CTGAACGGAGAGCTTTTATGCCTTATTGG
BhaB7_R208A_R	GGCTTCCTGCAATCGGGG

Table S2. MALDI-TOF MS data of CFE containing BhaA-Ala variants PCR fragments only or along with BhaB$_C^{Trp}$ plasmids as shown in Figure 2B and Figure S2.

Variants	Calcd. m/z of unmodified variants	Obsd. m/z of unmodified variants	Calcd. m/z of Trp-appended variants	Obsd. m/z of Trp-appended variants
BhaA-Ala-delete-1	2160.08	2160.13	2346.16	2346.14
BhaA-Ala- delete-2	1427.75	1428.10	1613.83	1614.16
BhaA-Ala- delete-3	4028.92	4029.29	4215.00	-
BhaA-Ala-(25-28A)	4351.12	4351.46	4537.20	4537.18
BhaA-Ala-(27-30A)	4371.09	4371.32	4557.17	4557.15
BhaA-Ala-(29-32A)	4369.15	4369.27	4555.22	4555.24
BhaA-Ala-(31-34A)	4384.11	4384.16	4570.19	4570.20
BhaA-Ala-(33-36A)	4460.09	4460.28	4646.17	-
BhaA-Ala-(37-40A)	4487.10	4487.13	4673.18	-
BhaA-Ala-P36A	4545.18	4545.96	4731.26	4731.04
BhaA-Ala-L37A	4529.14	4528.57	4715.23	4715.07
BhaA-Ala-L37F	4605.18	4604.97	4791.26	4791.13
BhaA-Ala-A38F	4647.23	4647.23	4833.31	4832.99
BhaA-Ala-L39A	4529.14	4528.39	4715.23	4715.29
BhaA-Ala-L39F	4605.18	4604.99	4791.26	4790.69
BhaA-Ala-A40Y	4663.22	4662.83	4849.30	4849.15
BhaA-Ala-A40F	4647.23	4646.95	4833.31	4832.91
BhaA-Ala-A40L	4613.24	4613.24	4799.32	4799.11
BhaA-Ala-A40G	4557.18	4557.16	4743.26	4742.99
BhaA-Ala-A40K	4628.25	4628.01	4814.33	4814.32
BhaA-Ala-A40E	4629.20	4628.78	4815.28	4815.08
BhaA-Ala-A40S	4587.19	4587.00	4773.27	4773.15
BhaA-Ala-A40W	4686.24	4686.17	4872.32	4872.38
BhaA-Ala-A40P	4597.21	4597.02	4783.29	4783.19

BhaA-Ala- extend-1	4926.38	4926.05	5112.46	5112.39
BhaA-Ala- extend-2	5153.54	5153.45	5339.62	5339.47
BhaA-Ala- extend-3	5397.61	5397.48	5554.69	5584.11

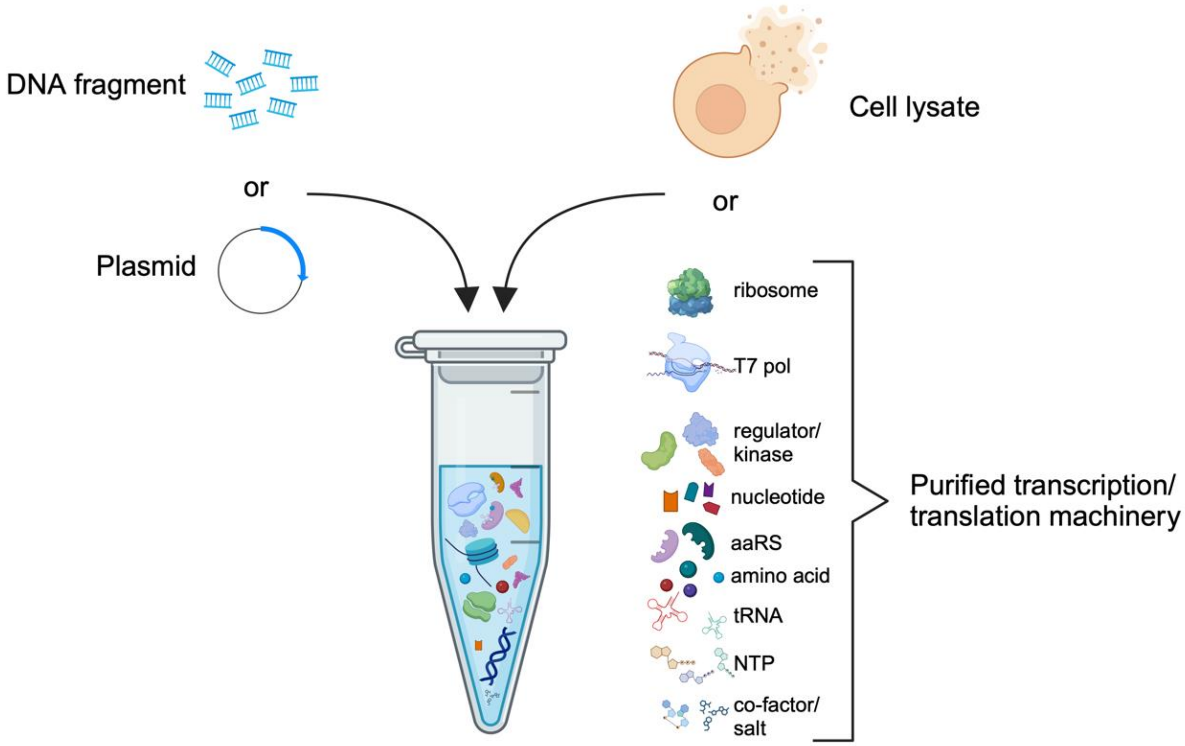

Figure S1. Protein production using a cell-free expression system. DNA fragments or plasmids containing genes of interest are added into the cell lysate or purified transcription/translation machinery. Key components in purified transcription/translation machinery are depicted, including ribosome, T7 polymerase, regulator/kinase (e.g., initiation factors, elongation factors, release factors, nucleoside-diphosphate kinase, creatine kinase, myokinase, pyrophosphatase), amino acids and their tRNA synthase as well as tRNA, NTP, and co-factors (1). The figure was created with BioRender.com.

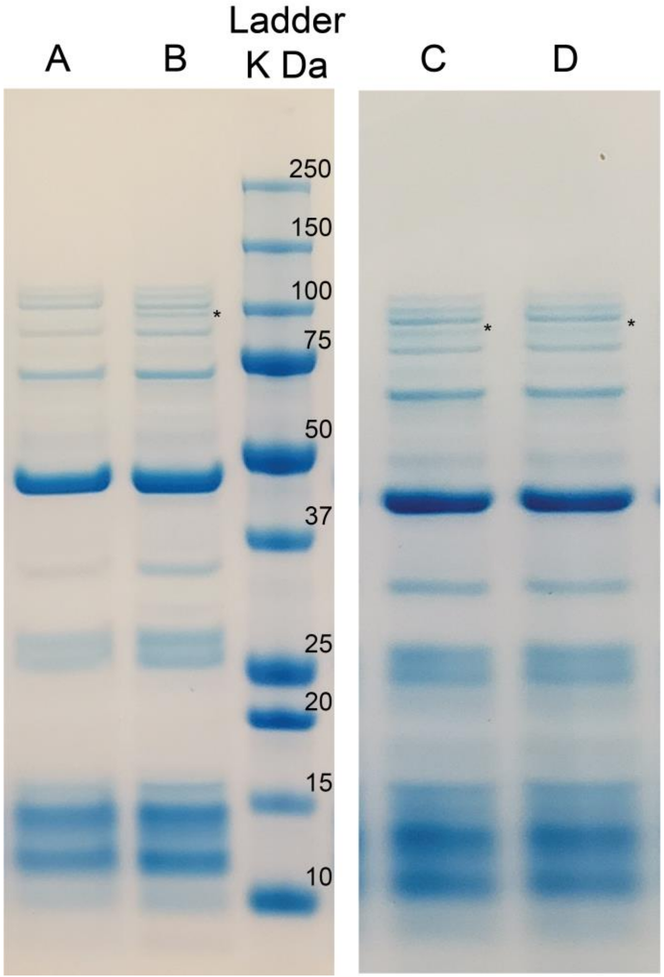

Figure S2. SDS-PAGE analysis of CFE reactions evaluating the expression of $\mathrm{BhaB_C^{Trp}}$ (MW=99 K Da) as indicated by an asterisk. (A) Control CFE reaction without DNA template. (B) CFE reaction containing the plasmid encoding $\mathrm{BhaB_C^{Trp}}$. (C) CFE reaction containing 160 ng of plasmid encoding BhaA-Ala and 60 ng of plasmid encoding $\mathrm{BhaB_C^{Trp}}$. (D) CFE reaction containing 160 ng of BhaA-Ala-encoding plasmid and 150 ng of $\mathrm{BhaB_C^{Trp}}$-encoding plasmid. Although the expressed enzyme band is faint in lanes C and D, MALDI-TOF MS analysis showed full attachment of Trp to the peptide substrate.

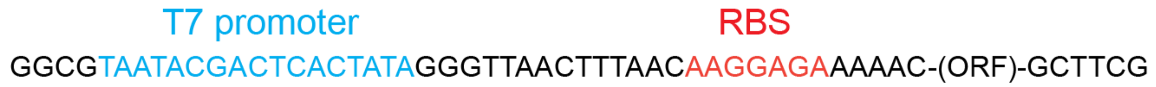

Figure S3. PCR fragment design for expression of Bha-Ala variants. This design only contains the T7 promoter, RBS and ORF linked with spacers. In this design, the ORF directly starts with the DNA encoding the protein of interest without tag.

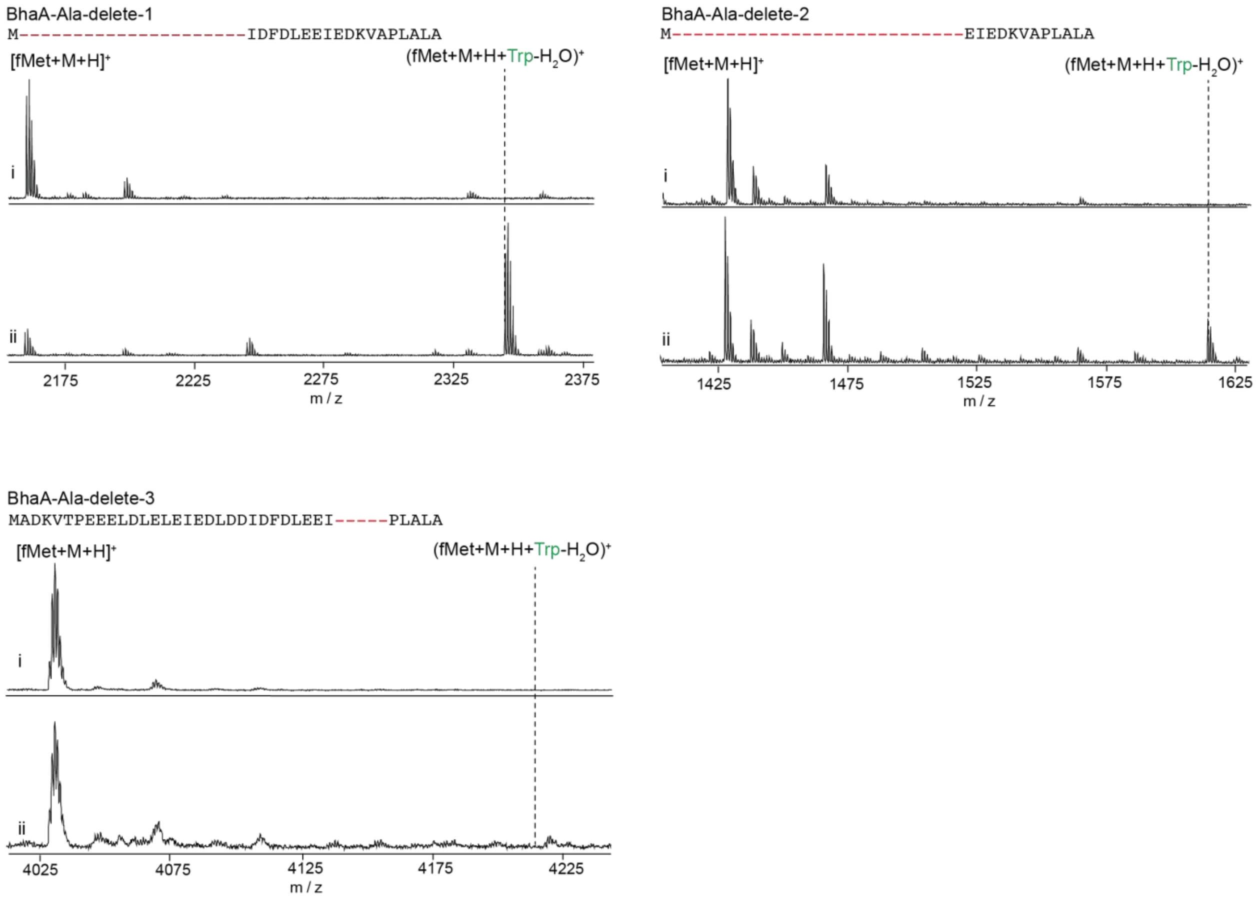

Figure S4. MALDI-TOF MS of CFE reactions containing (i) shortened BhaA-Ala variants generated from PCR fragments encoding the designed genes, or (ii) shortened BhaA-Ala variants encoded on the PCR fragments along with $BhaB_C^{Trp}$ plasmids. *m/z* values are listed in Table S2.

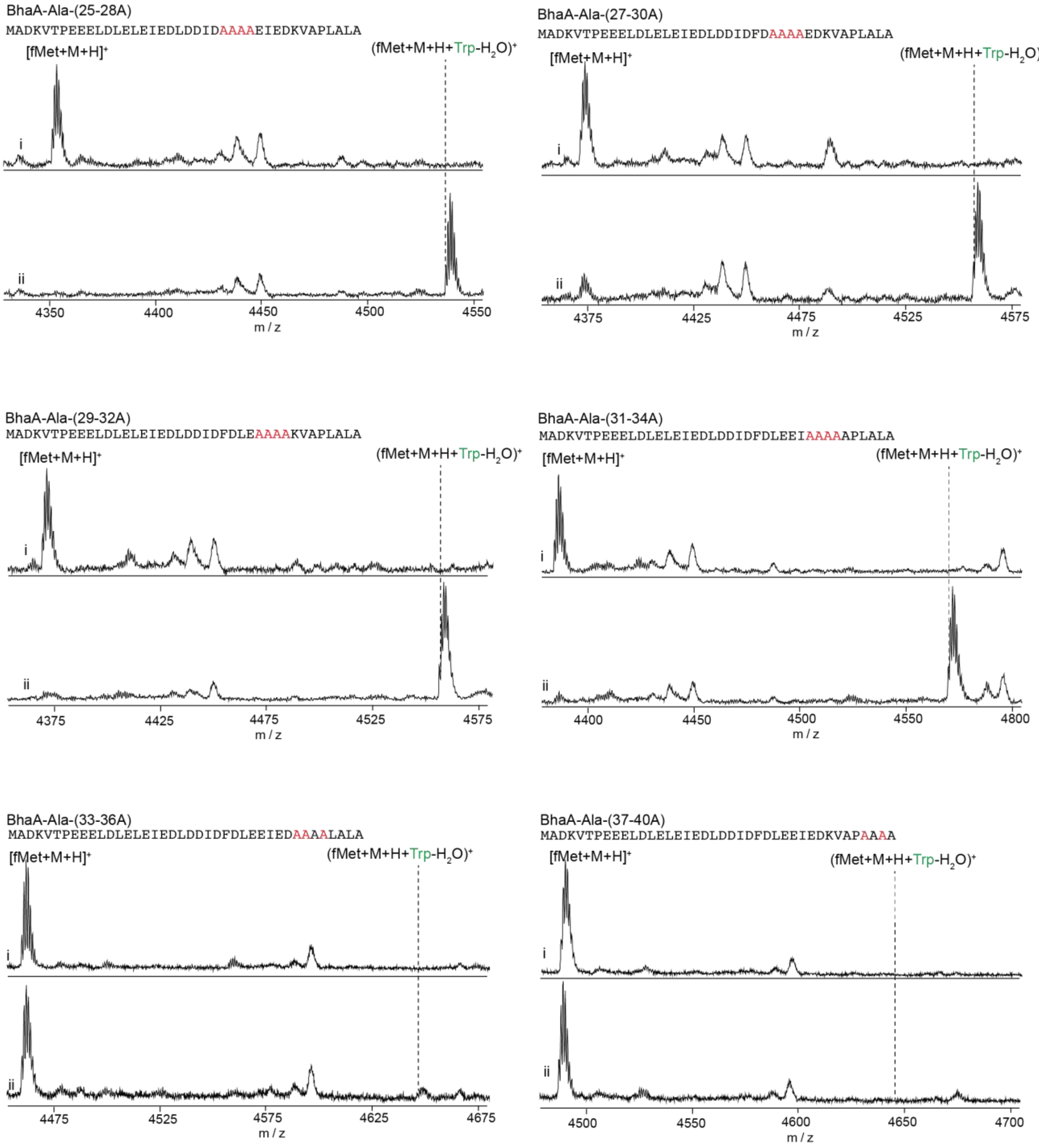

Figure S5. MALDI-TOF MS of CFE reactions containing (i) BhaA-Ala variants with Ala block scanning generated from PCR fragments encoding the designed genes, or (ii) BhaA-Ala variants with Ala block scanning encoded on the PCR fragments along with $BhaB_C^{Trp}$ plasmids. *m/z* values are listed in Table S2.

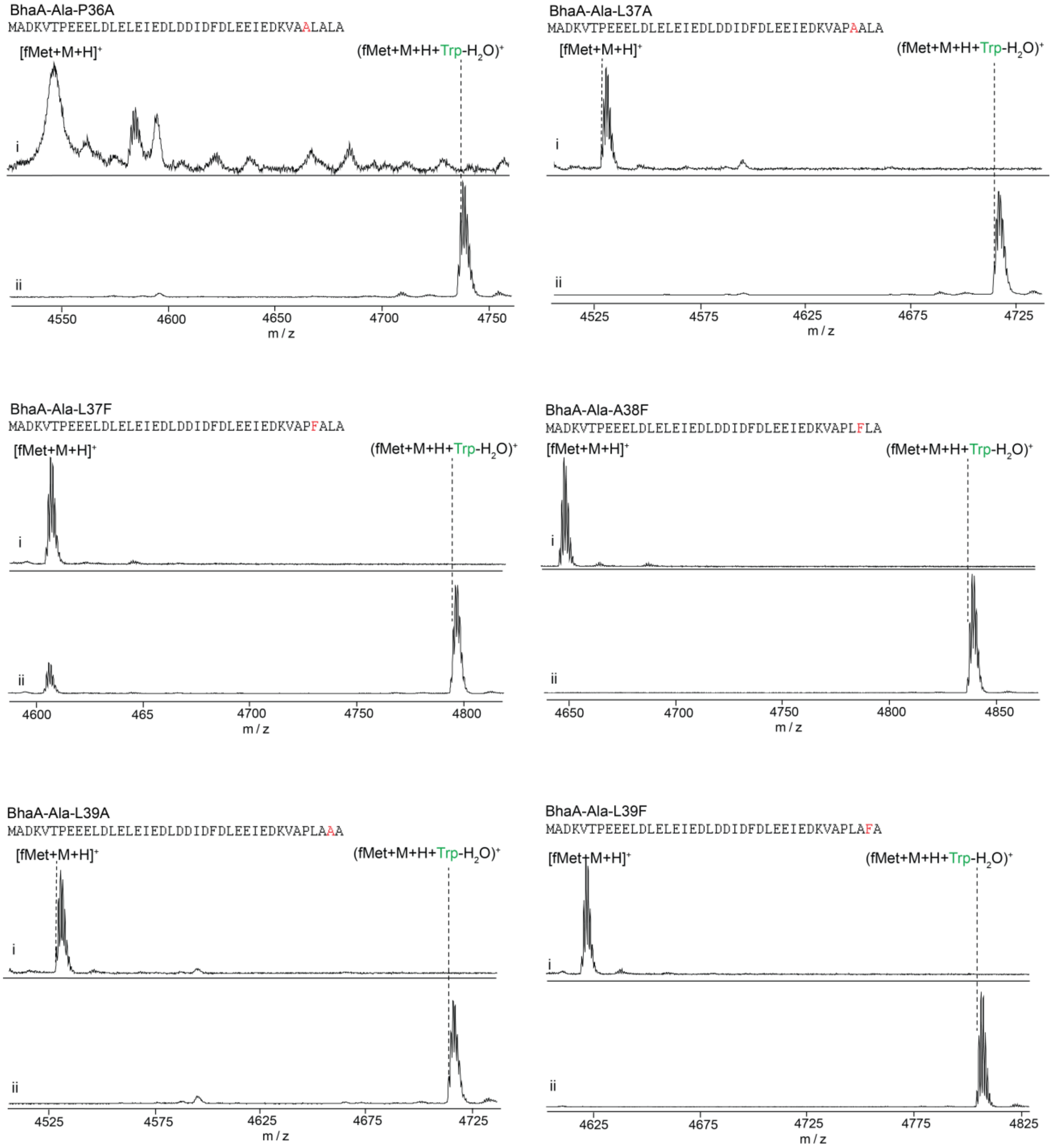

Figure S6. MALDI-TOF MS of CFE reactions containing (i) BhaA-Ala variants with single site mutation from Pro36 to Leu39 generated from PCR fragments encoding the designed genes, or (ii) BhaA-Ala variants with single site mutation from Pro36 to Leu39 encoded on the PCR fragments along with $\mathrm{BhaB_C^{Trp}}$ plasmids. *m/z* values are listed in Table S2.

BhaA-Ala-A40G
MADKVTPEEELDLELEIEDLDDIDFDLEEIEDKVAPLALG

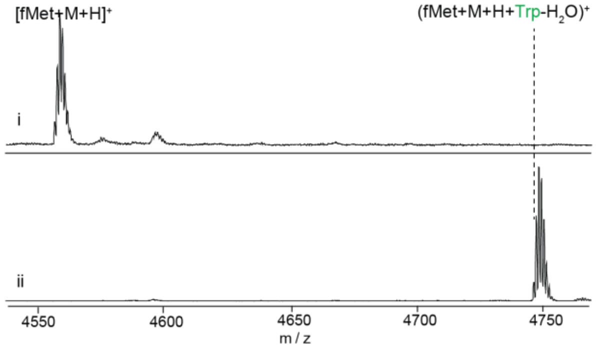

BhaA-Ala-A40K
MADKVTPEEELDLELEIEDLDDIDFDLEEIEDKVAPLALK

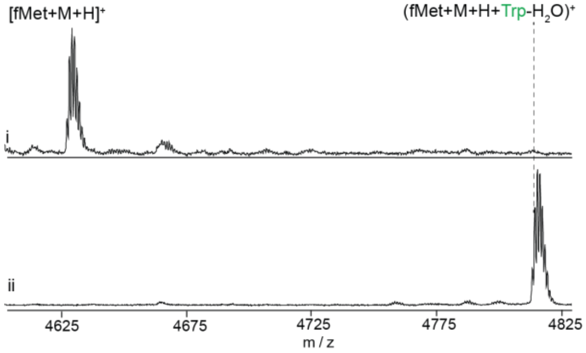

BhaA-Ala-A40E
MADKVTPEEELDLELEIEDLDDIDFDLEEIEDKVAPLALE

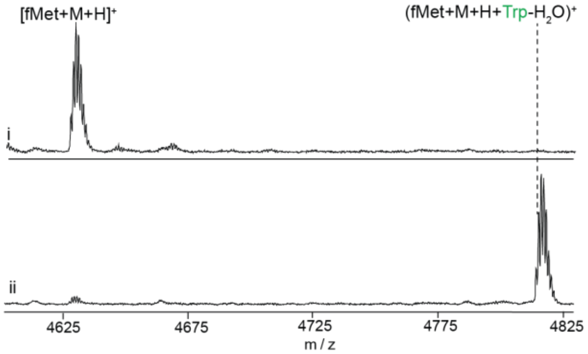

BhaA-Ala-A40S
MADKVTPEEELDLELEIEDLDDIDFDLEEIEDKVAPLALS

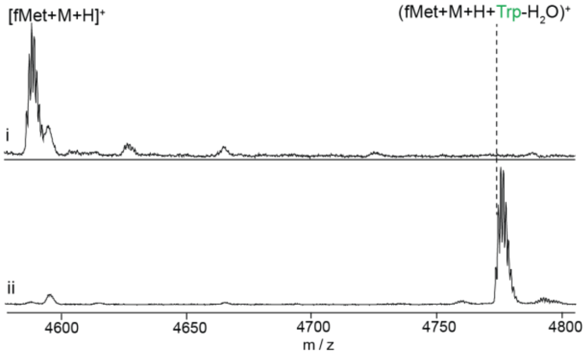

BhaA-Ala-A40W
MADKVTPEEELDLELEIEDLDDIDFDLEEIEDKVAPLALW

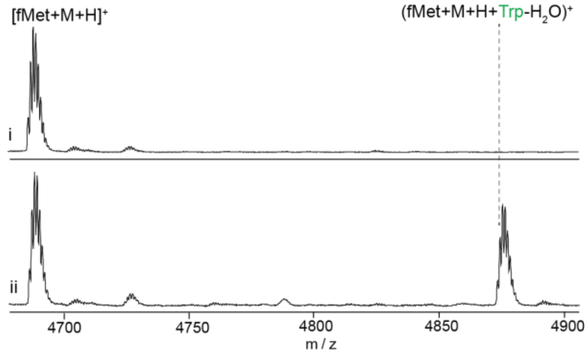

BhaA-Ala-A40P
MADKVTPEEELDLELEIEDLDDIDFDLEEIEDKVAPLALP

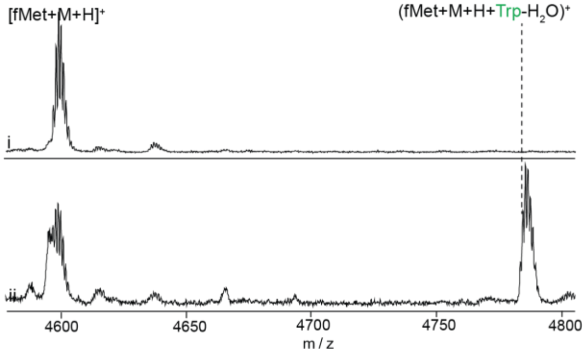

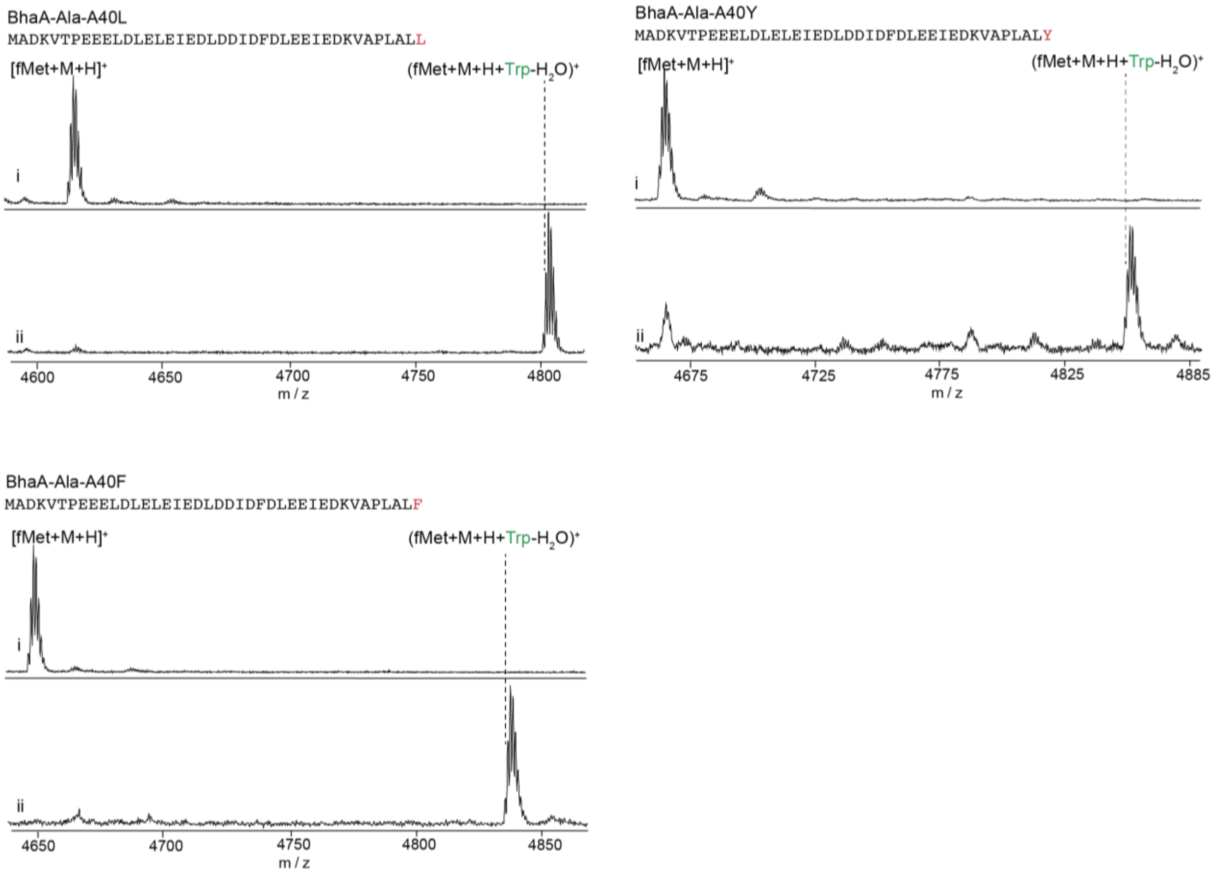

Figure S7. MALDI-TOF MS of CFE reactions containing (i) BhaA-Ala variants with single site mutation of Ala40 generated from PCR fragments encoding the designed genes, or (ii) BhaA-Ala variants with single site mutation of Ala40 encoded on the PCR fragments along with $BhaB_C^{Trp}$ encoded on a plasmid. *m/z* values are listed in Table S2.

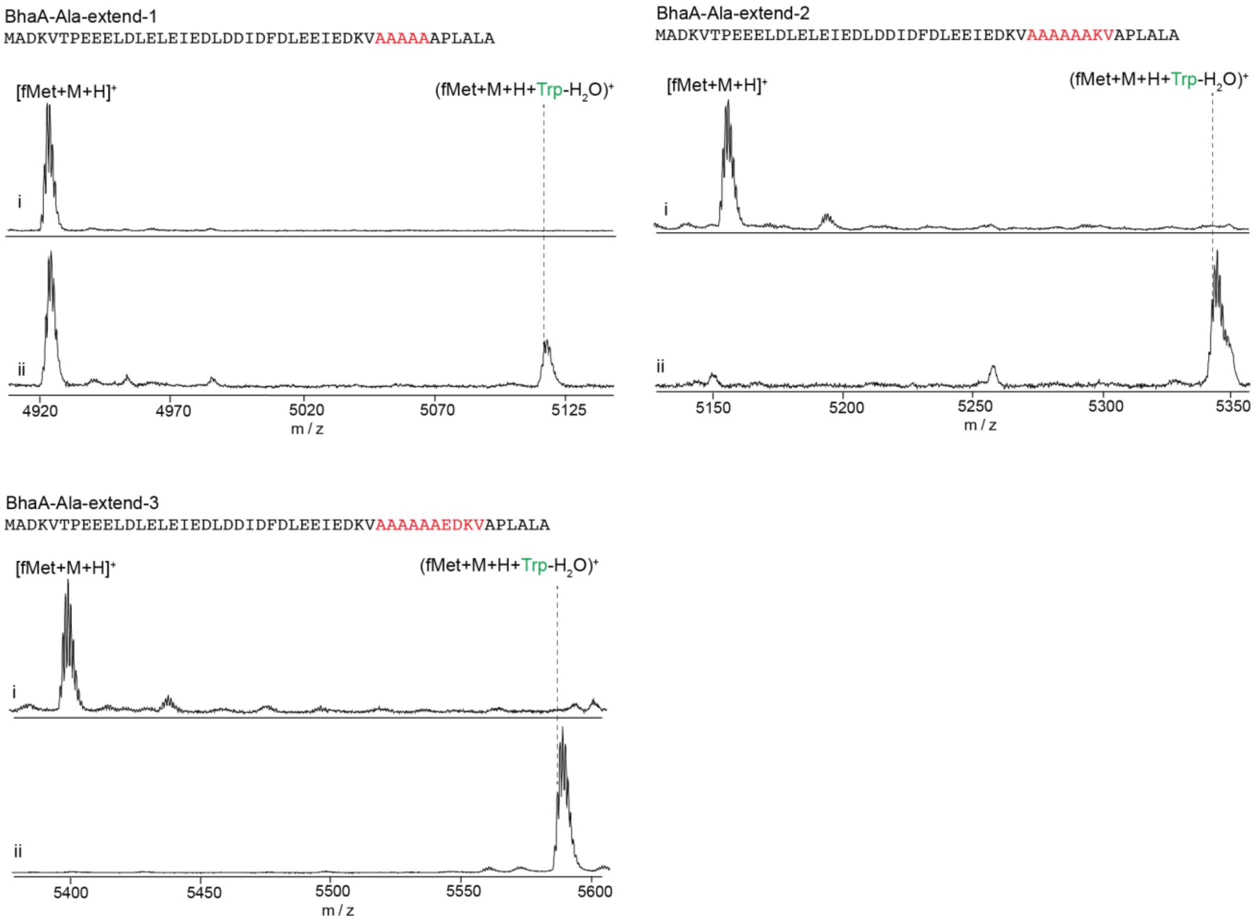

Figure S8. MALDI-TOF MS of CFE reactions containing (i) lengthened BhaA-Ala variants generated from PCR fragments encoding the designed genes, or (ii) lengthened BhaA-Ala variants encoded on the PCR fragments along with $\mathrm{BhaB}_{\mathrm{C}}^{\mathrm{Trp}}$ plasmids. *m/z* values are listed in Table S2.

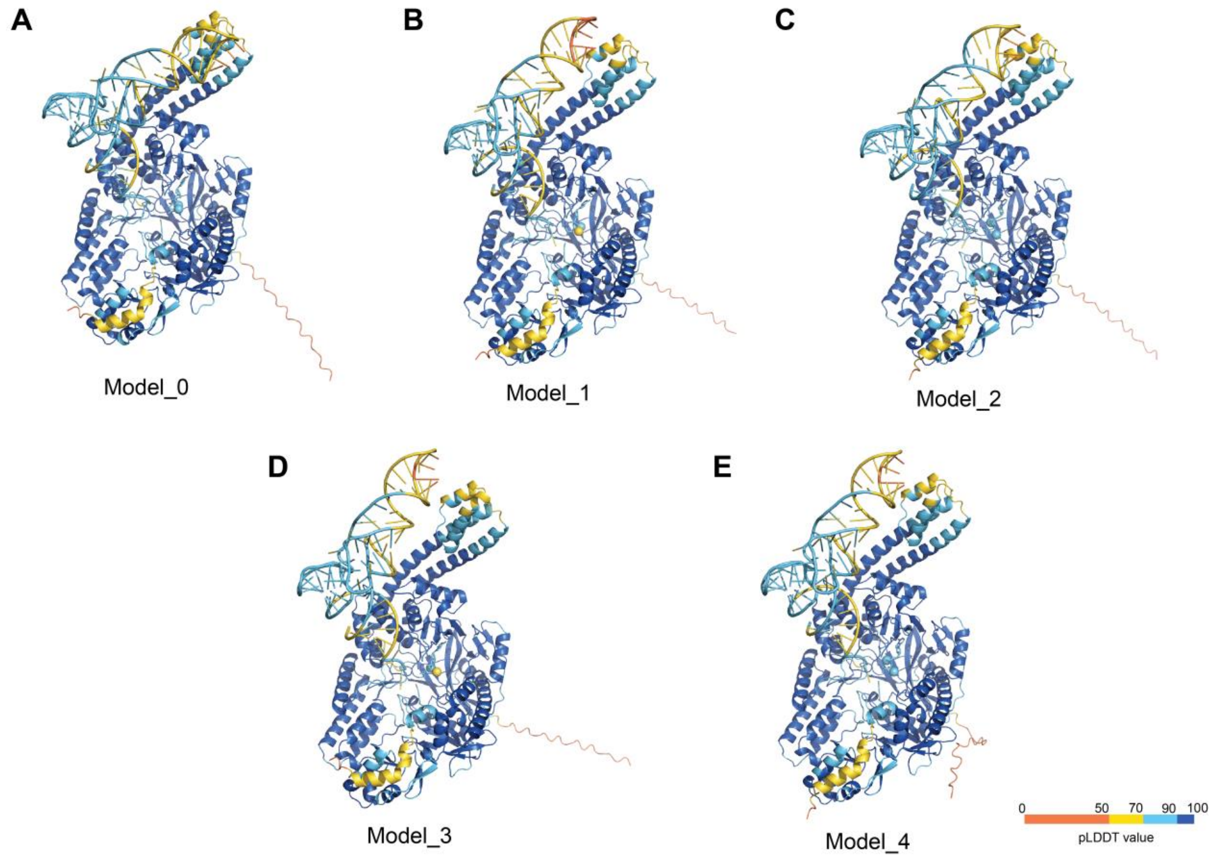

Figure S9. Top five models output of AlphaFold3 prediction of the complex between the native $BhaB_C^{Trp}$, $tRNA^{Trp}$, BhaA-Ala, ATP, and Mg^{2+} ions. Coloring is by pLDDT values.

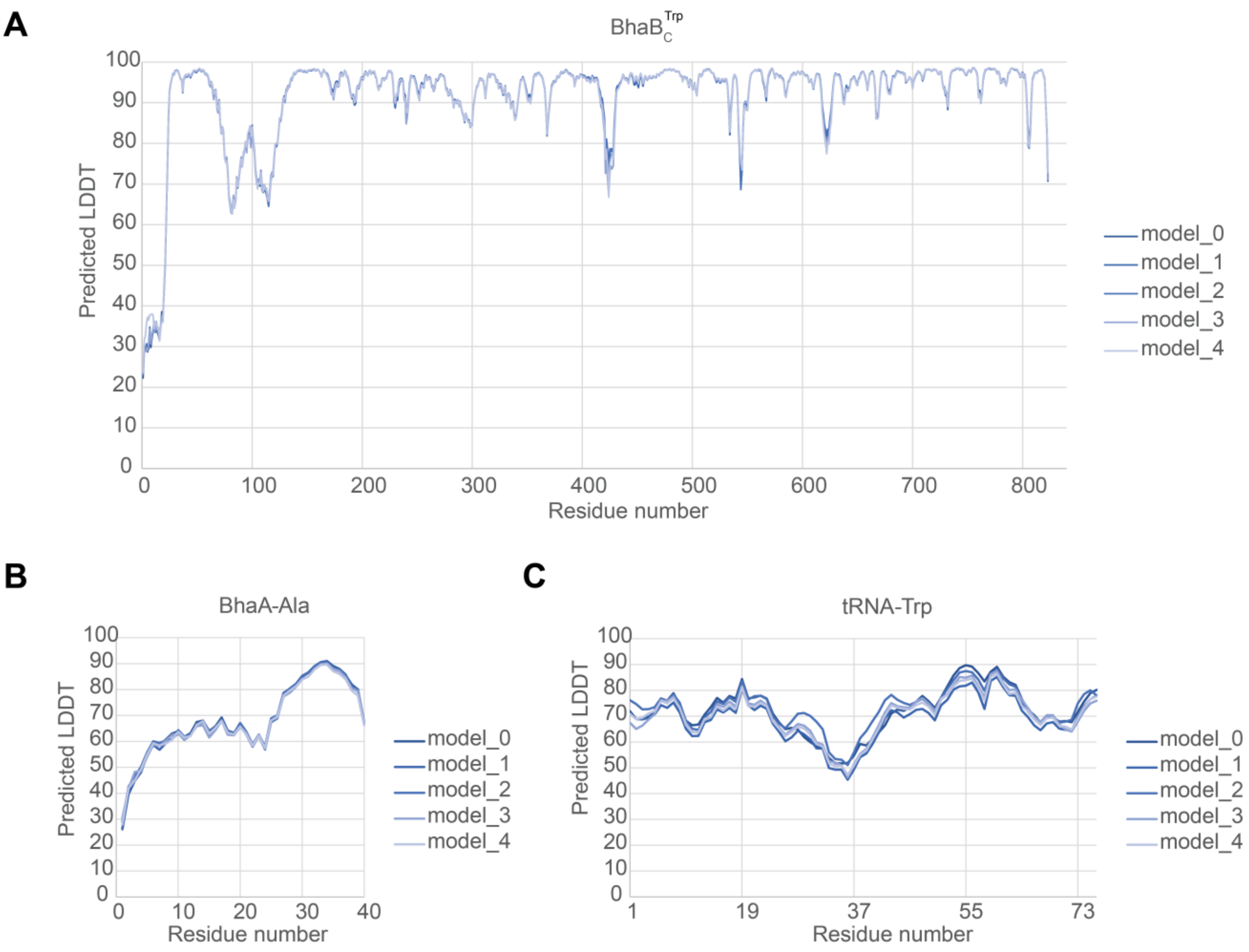

Figure S10. Predicted LDDT scores vs residue number obtained for the top five models retrieved from AlphaFold3 prediction of the complex between BhaB$_C^{Trp}$, tRNA^{Trp}, BhaA-Ala, ATP, and Mg^{2+} ions.

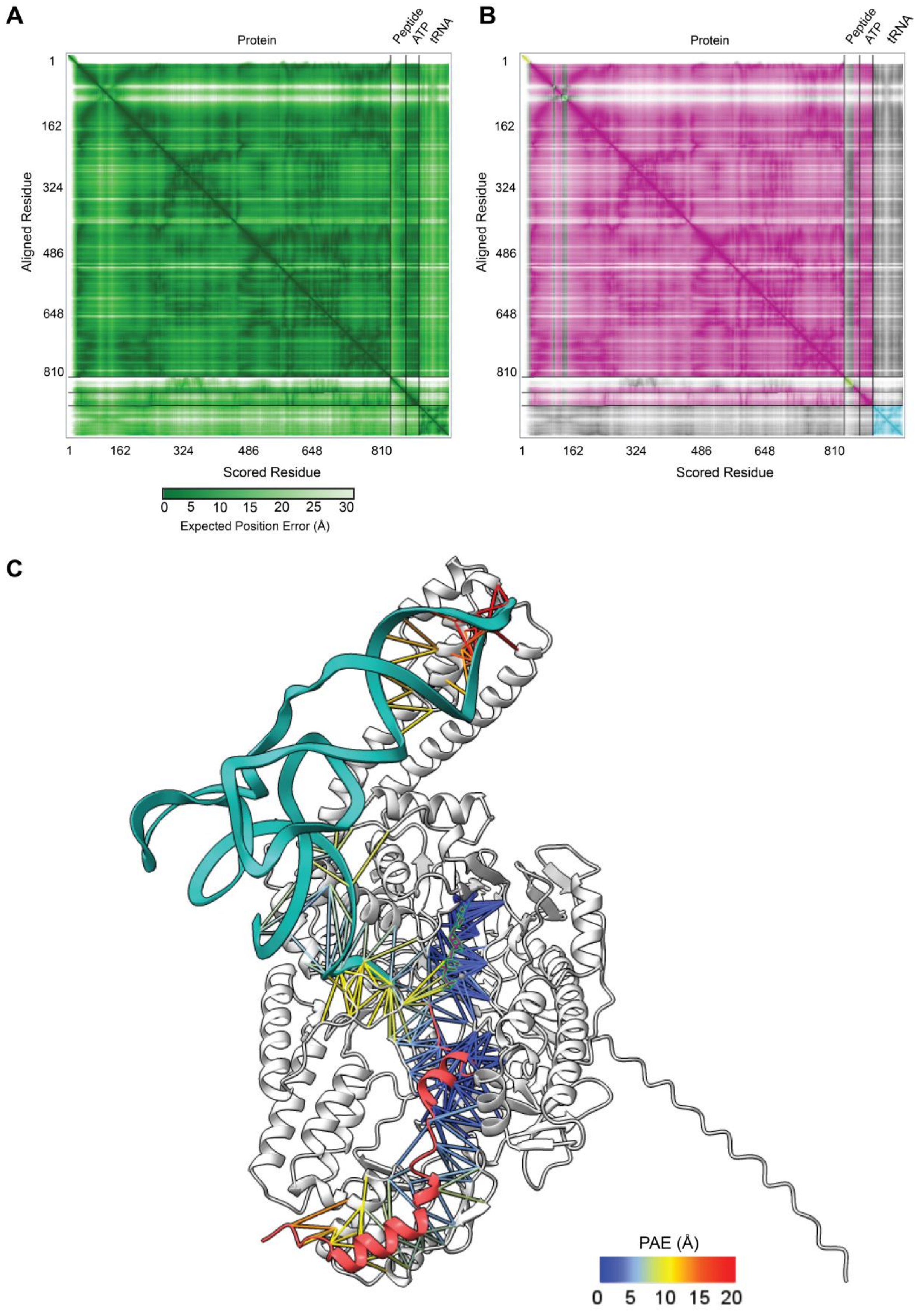
A
B
C
Protein
Peptide
ATP
tRNA
Aligned Residue
Scored Residue
1
162
324
486
648
810
0 5 10 15 20 25 30
Expected Position Error (Å)
PAE (Å)
0 5 10 15 20

Figure S11. A) Predicted Aligned Error (PAE) plot for the AlphaFold3 prediction of the complex between $BhaB_C^{Trp}$, $tRNA^{Trp}$, BhaA-Ala, and ATP. Dark green are regions of high confidence, light green regions of low confidence. B) PAE domains for the AlphaFold3 prediction based on the relative residue error obtained using ChimeraX 1.8 (11). The C-terminus of BhaA-Ala and ATP are colored similarly indicating the PAE value is less than 5 Å. C) Interacting residues or bases involving $BhaB_C^{Trp}$, substrate peptide (BhaA-Ala in salmon), ATP (purple), and $tRNA^{Trp}$ (light blue) are shown in sticks colored by PAE value (11). A colored scale bar indicating the PAE values for each contact pair is provided.

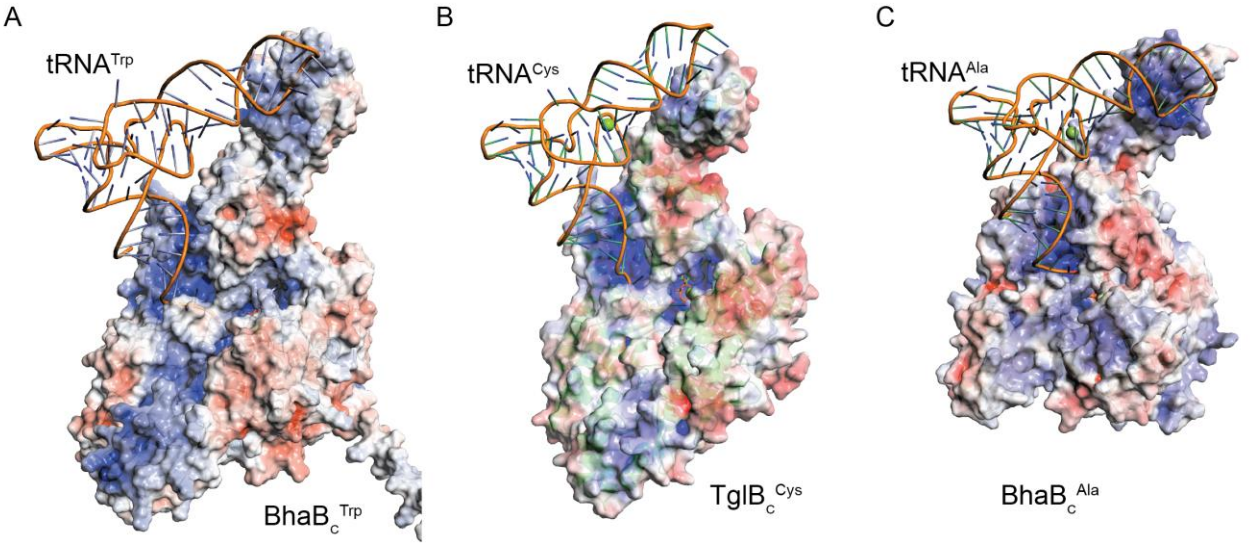

Figure S12. AlphaFold3 predictions of the cognate tRNA binding modes to the three PEARLs discussed in the main text; ATP and substrate peptide were also included but are not shown for clarity. In all cases, the tRNAs engage in a similar fashion with the 3'-end in the active site and the anticodon loop interacting with a coiled-coil domain of the enzymes. The PEARLs are shown with electrostatic density surface potentials (blue positive, red negative, white hydrophobic) demonstrating that the predicted contacts with the tRNA involve positively charged patches on the enzymes.

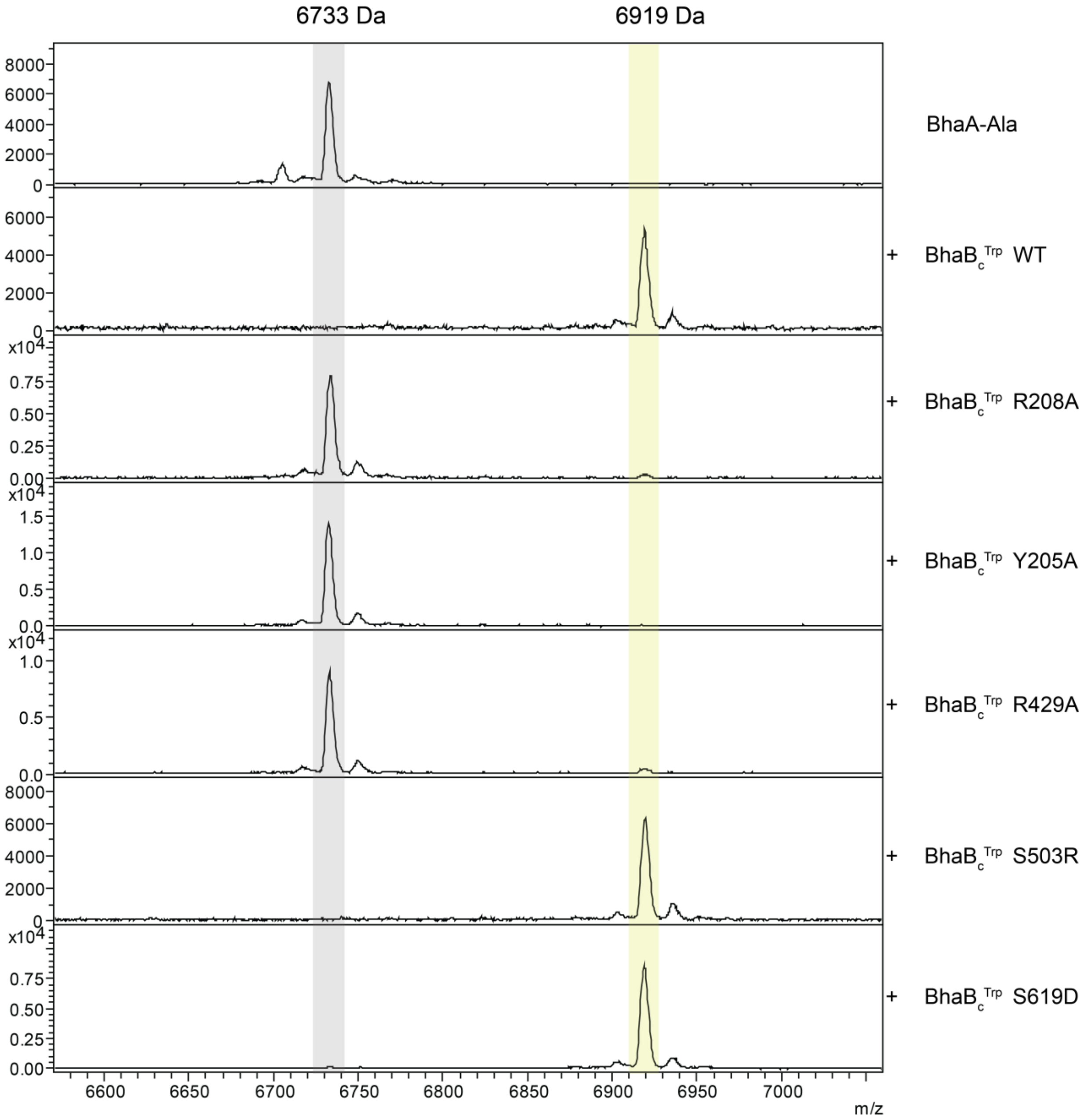

Figure S13. MALDI-TOF MS analysis of reactions of $BhaB_C^{Trp}$ variants with BhaA-Ala.

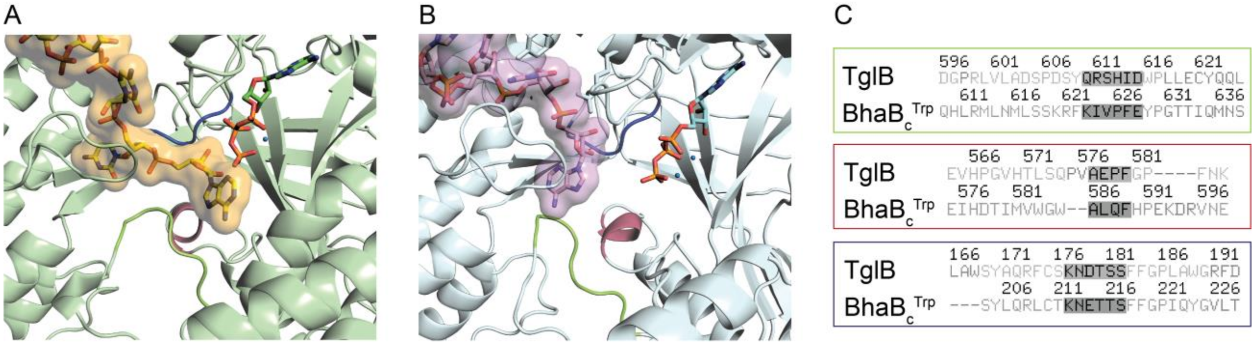

Figure S14. Regions near the 3’-end of the tRNA (A76) that may be involved in recognition of the amino acid attached to the tRNA. (A) AlphaFold3 model of the interaction of TglB with $tRNA^{Cys}$. (B) AlphaFold3 model of the interaction of $BhaB_C^{Trp}$ with $tRNA^{Trp}$. (C) Amino acid sequences corresponding to the colored regions of the enzymes that are near the ribose of A76.

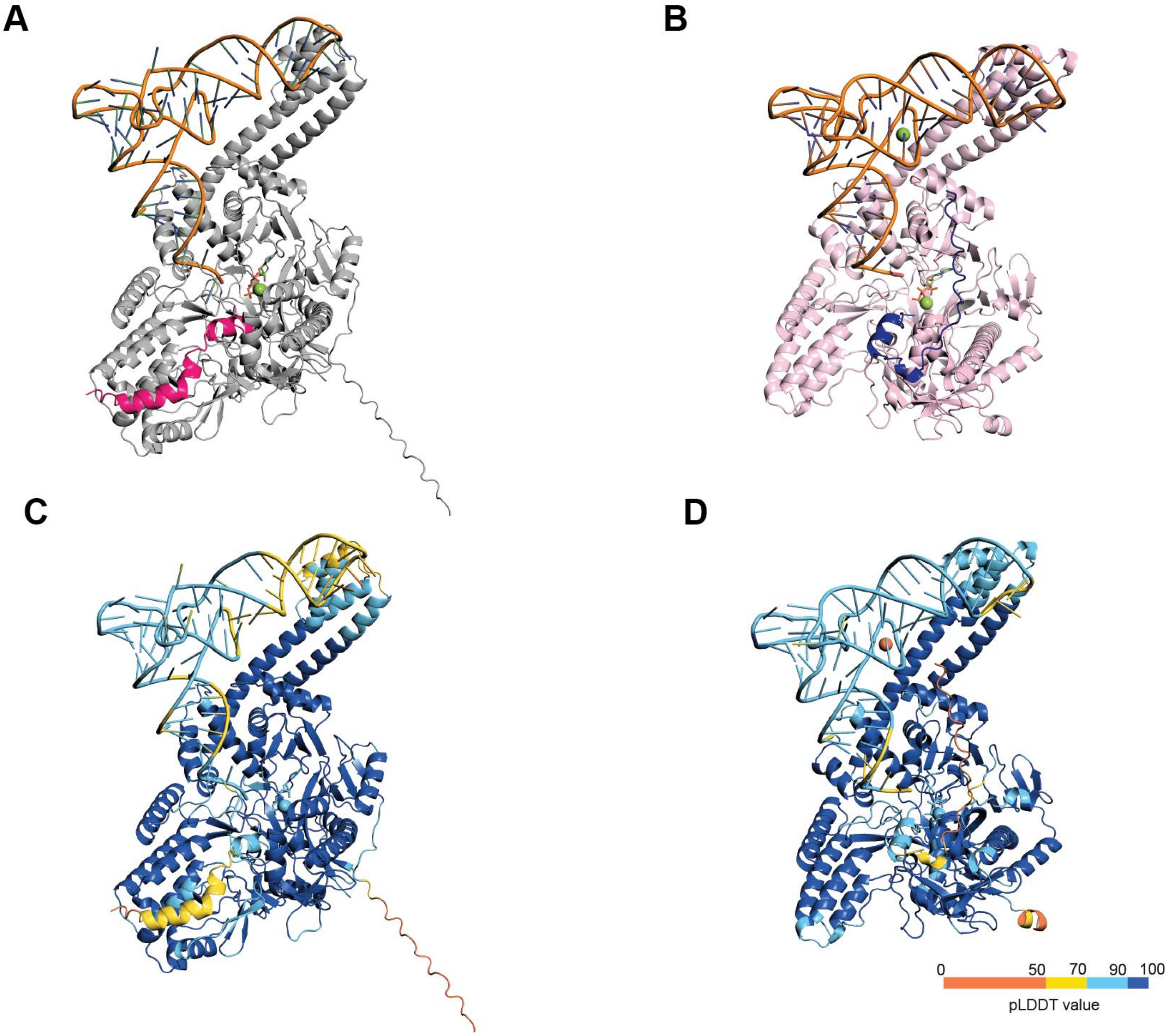

Figure S15. AlphaFold3 models of (A) BhaA-Ala (pink) binding to $BhaB_C^{Trp}$:tRNA^{Trp}:ATP and (B) BhaA (blue) binding to $BhaB_C^{Ala}$:tRNA^{Ala}:ATP. Both peptides are predicted to have their C-terminal carboxylates close to the ATP γ-phosphate and have a helical structure near the C-terminus, but otherwise the conformations are quite different. We note that the pLDDT values are low for large parts of the substrate peptides (panels C and D). Neither peptide is predicted to engage the RRE.

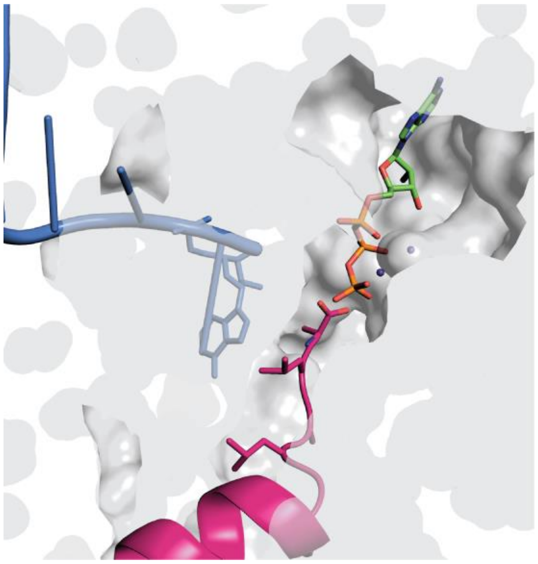

Figure S16. AlphaFold3 predicted secondary structure for BhaA-Ala showing a cavity available near the side chain of the C-terminal amino acid of the substrate peptide. BhaA-Ala is shown in dark pink.

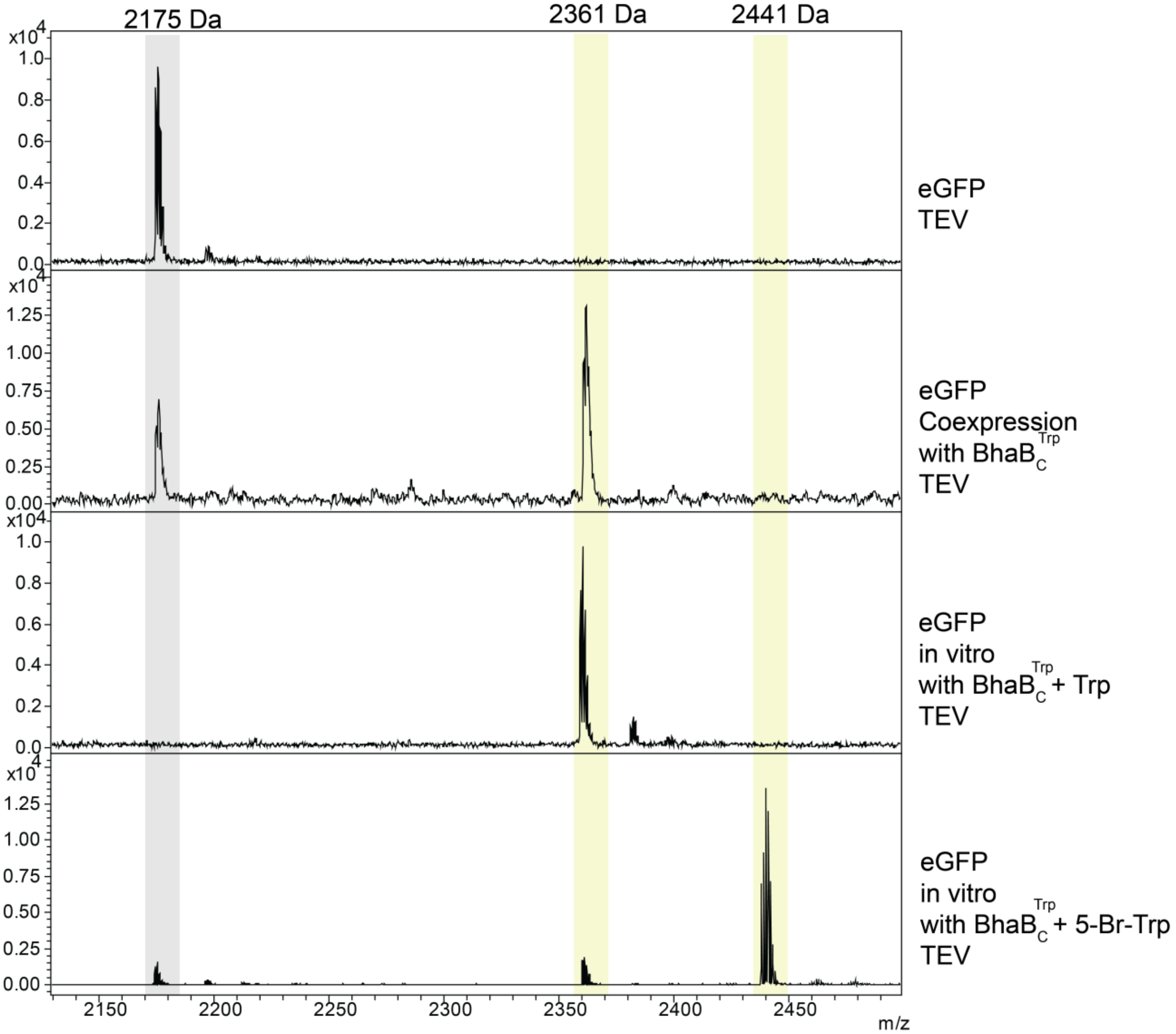

Figure S17. MALDI-TOF MS analysis of *in vivo* and *in vitro* experiments using BhaB$_C^{Trp}$-mediated Trp conjugation to the LEEIEDKVAPLALA-tagged eGFP C-terminus. Top panel: purified tagged eGFP digested with TEV. Second panel: TEV digested product of co-expression of tagged eGFP and BhaB$_C^{Trp}$. Third panel: TEV digested product of *in vitro* reaction of tagged eGFP and BhaB$_C^{Trp}$ in the presence of Trp. Fourth panel: TEV digested product of *in vitro* reaction of eGFP and BhaB$_C^{Trp}$ in the presence of 5-Br-Trp. The data shown in the fourth panel is after most Trp was removed from TrpRS. For data before removal of Trp, see Fig. S20.

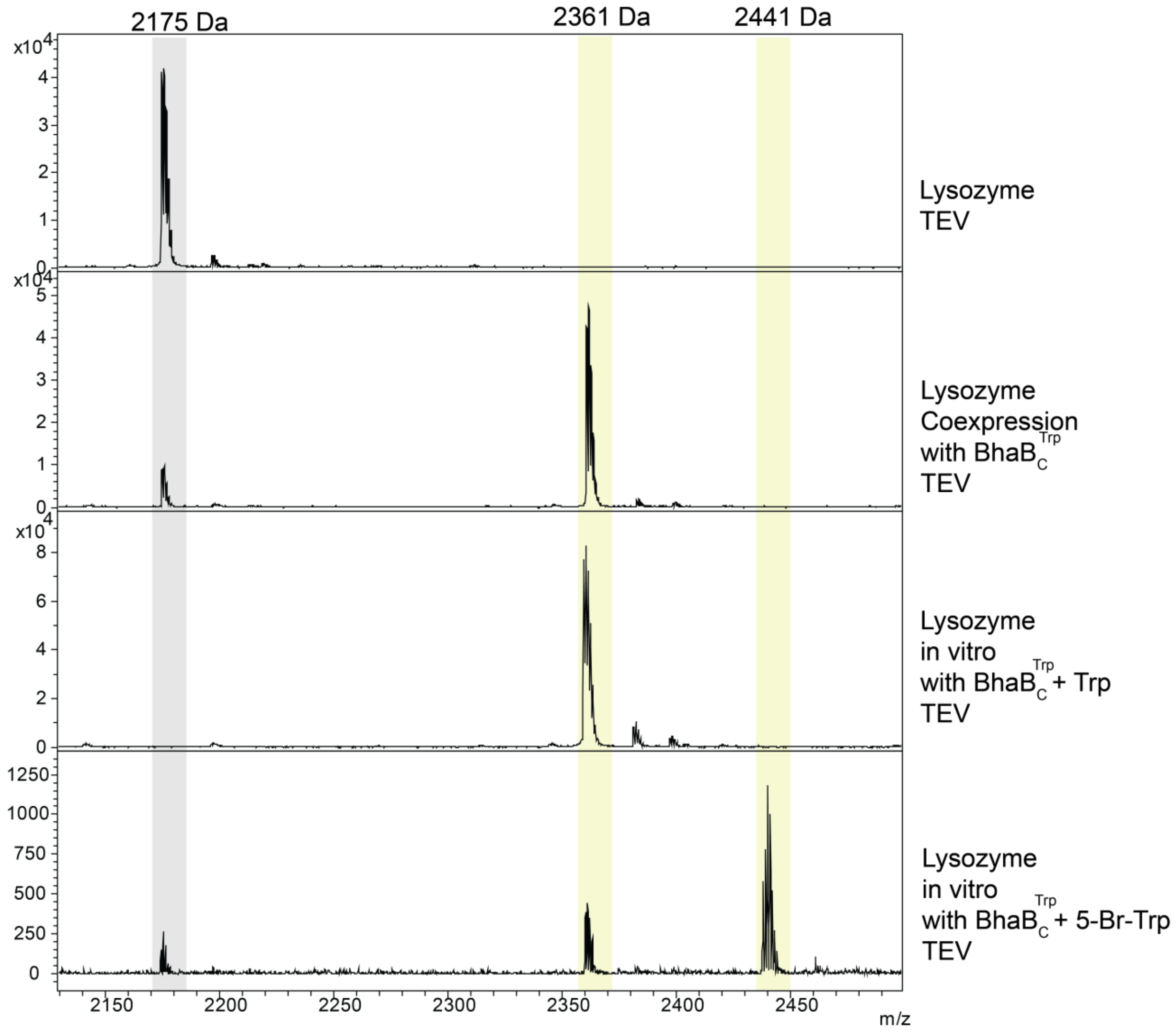

Figure S18. MALDI-TOF MS analysis of BhaB$_C^{Trp}$ *in vivo* an *in vitro* experiments for Trp incorporation to LEEIEDKVAPLALA tagged lysozyme C-terminus. Top panel: purified tagged lysozyme digested with TEV. Second panel: TEV digested product of co-expression of lysozyme and BhaB$_C^{Trp}$. Third panel: TEV digested product of *in vitro* reaction of lysozyme and BhaB$_C^{Trp}$ in the presence of Trp. Fourth panel: TEV digested product of *in vitro* reaction of lysozyme and BhaB$_C^{Trp}$ in the presence of 5-Br-Trp. The data shown in the fourth panel is after most Trp was removed from TrpRS. For data before removal of Trp, see Fig. S20.

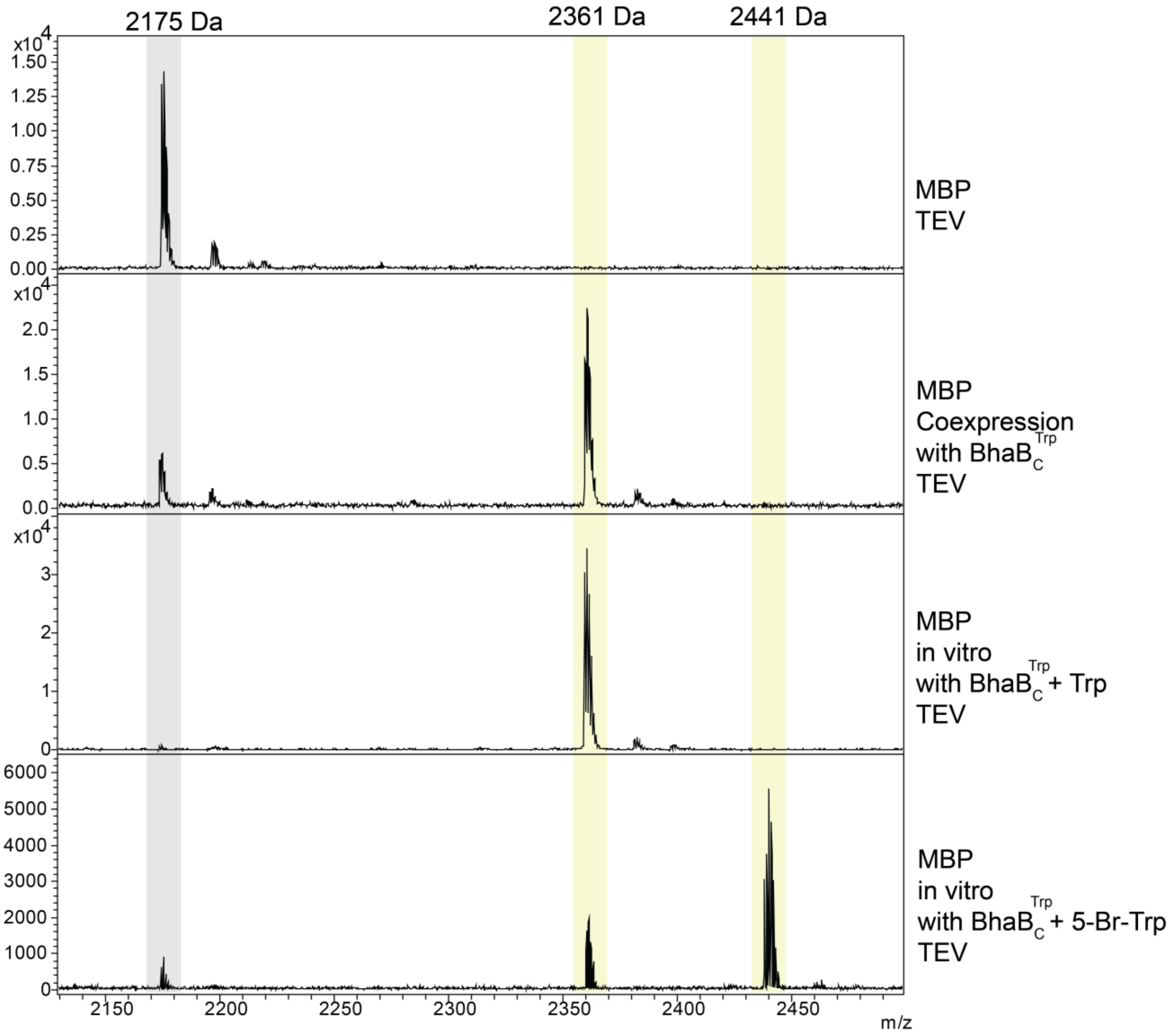

Figure S19. MALDI-TOF MS analysis of $BhaB_C^{Trp}$ *in vivo* an *in vitro* experiments for Trp incorporation to LEEIEDKVAPLALA tagged MBP C-terminus. Top panel: purified tagged MBP digested with TEV. Second panel: TEV digested product of co-expression of MBP and $BhaB_C^{Trp}$. Third panel: TEV digested product of *in vitro* reaction of MBP and $BhaB_C^{Trp}$ in the presence of Trp. Fourth panel: TEV digested product of *in vitro* reaction of MBP and $BhaB_C^{Trp}$ in the presence of 5-Br-Trp. The data shown in the fourth panel is after most Trp was removed from TrpRS. For data before removal of Trp, see Fig. S20.

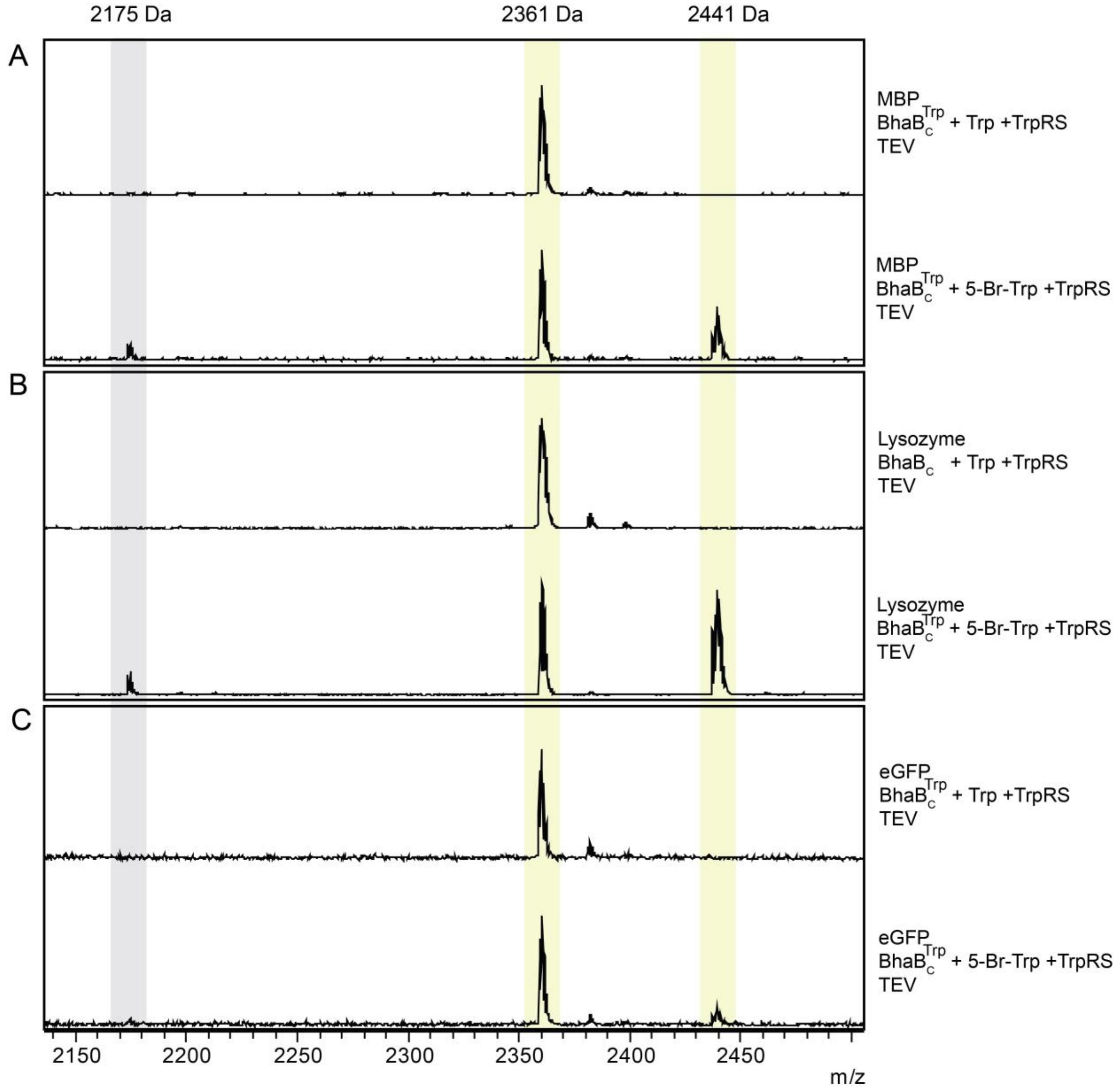

Figure S20. MALDI-TOF MS analysis of $\mathrm{BhaB_C^{Trp}}$ *in vitro* reactions using *E. coli* TrpRS in combination with L-Trp (top) and L-5-Br-Trp (bottom panel) for each enzyme fusion construct. (A) MBP, (B) lysozyme, and (C) eGFP. As previously observed, TrpRS copurifies with Trp-AMP, which can be removed with an additional buffer exchange step using L-5-Br-Trp and ATP leading to improved incorporation of 5-Br-Trp as shown in the bottom panels of Fig. S17-S19.

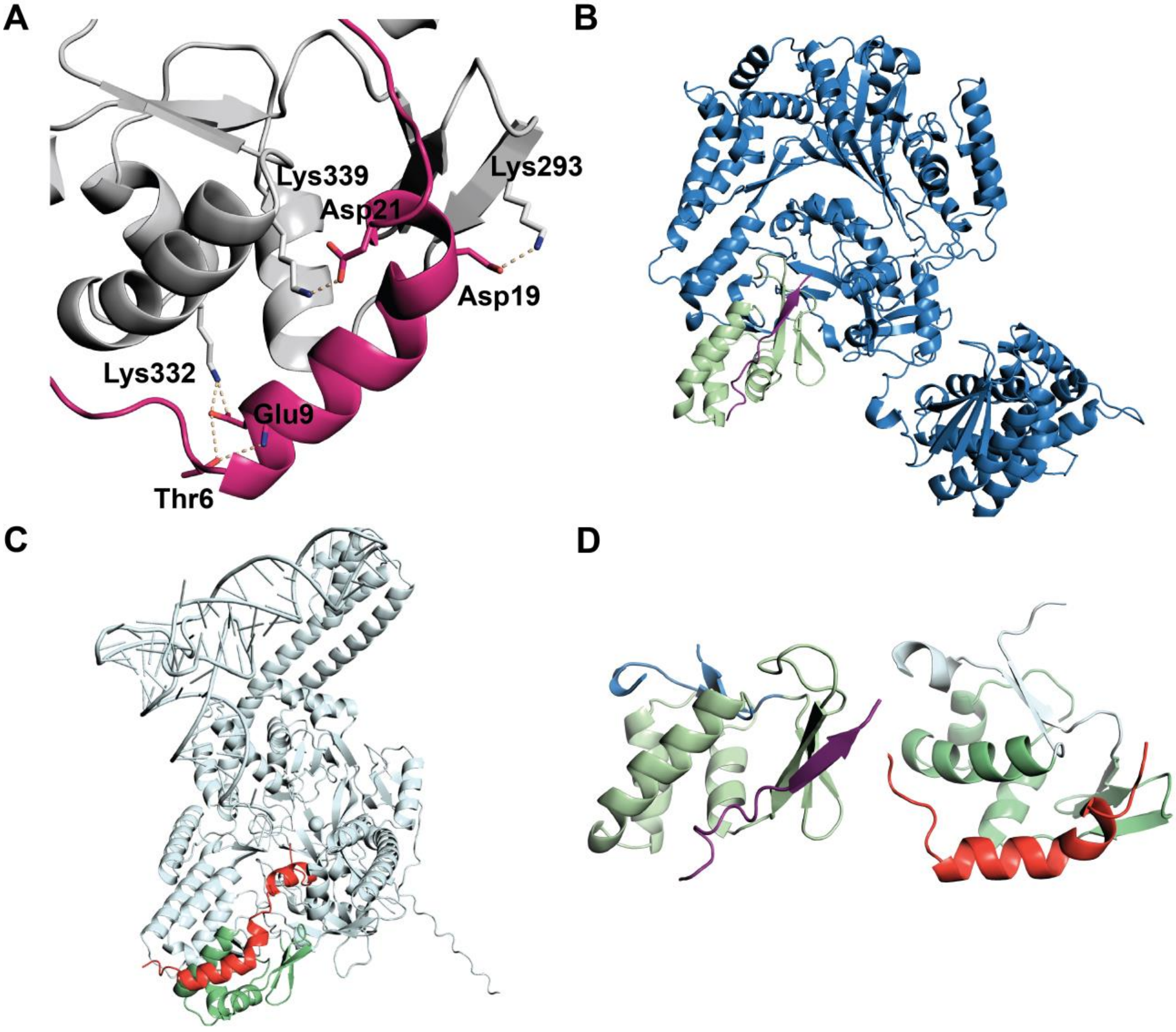

Figure S21. A) AlphaFold3 predicted secondary structure and binding of the N-terminal helix of the substrate BhaA_Ala to $BhaB_C^{Trp}$. B) NisB crystal structure obtained in complex with the leader region of the substrate peptide NisA bound to the RRE region of NisB. C) AlphaFold3 overall predicted binding of the substrate BhaA_Ala to the putative RRE region of PEARL $BhaB_C^{Trp}$, BhaA_Ala is shown in red and the RRE region in pastel green. D) Side comparison of the RRE regions (in pastel green) of NisB (left) and of the predicted structure of $BhaB_C^{Trp}$ (right) bound to the leader region of substrate NisA (purple) versus the N-terminal predicted secondary structure of BhaA_Ala (red).

References

1. Shimizu Y, *et al.* (2001) Cell-free translation reconstituted with purified components. *Nat. Biotechnol.* 19(8):751-755.
2. Daniels PN, *et al.* (2022) A biosynthetic pathway to aromatic amines that uses glycyl-tRNA as nitrogen donor. *Nat. Chem.* 14(1):71-77.
3. Ramos Figueroa J, Zhu L, & van der Donk WA (2024) Unexpected transformations during pyrroloiminoquinone biosynthesis. *J. Am. Chem Soc.* 146(20):14235-14245.
4. Xiang M, *et al.* (2023) An asymmetric structure of bacterial TrpRS supports the half-of-the-sites catalytic mechanism and facilitates antimicrobial screening. *Nucleic Acids Res.* 51(9):4637-4649.
5. Burkhart BJ, Hudson GA, Dunbar KL, & Mitchell DA (2015) A prevalent peptide-binding domain guides ribosomal natural product biosynthesis. *Nat. Chem. Biol.* 11(8):564-570.
6. Chekan JR, Ongpipattanakul C, & Nair SK (2019) Steric complementarity directs sequence promiscuous leader binding in RiPP biosynthesis. *Proc. Natl. Acad. Sci. U. S. A.* 116(48):24049-24055.
7. Montalbán-López M, *et al.* (2021) New developments in RiPP discovery, enzymology and engineering. *Nat. Prod. Rep.* 38(1):130-239.
8. Ortega MA, *et al.* (2015) Structure and mechanism of the tRNA-dependent lantibiotic dehydratase NisB. *Nature* 517(7535):509-512.
9. Bothwell IR, *et al.* (2019) Characterization of glutamyl-tRNA-dependent dehydratases using nonreactive substrate mimics. *Proc. Natl. Acad. Sci. U. S. A.* 116(35):17245-17250.
10. Davis KM, *et al.* (2017) Structures of the peptide-modifying radical SAM enzyme SuiB elucidate the basis of substrate recognition. *Proc. Natl. Acad. Sci. USA* 114(39):10420-10425.
11. Meng EC, *et al.* (2023) UCSF ChimeraX: Tools for structure building and analysis. *Protein Sci.* 32(11):e4792.
